# Deoxyinosine triphosphate induces MLH1/PMS2- and p53-dependent cell growth arrest and DNA instability in mammalian cells

**DOI:** 10.1038/srep32849

**Published:** 2016-09-13

**Authors:** Yasuto Yoneshima, Nona Abolhassani, Teruaki Iyama, Kunihiko Sakumi, Naoko Shiomi, Masahiko Mori, Tadahiro Shiomi, Tetsuo Noda, Daisuke Tsuchimoto, Yusaku Nakabeppu

**Affiliations:** 1 Division of Neurofunctional Genomics, Department of Immunobiology and Neuroscience, Medical Institute of Bioregulation, Kyushu University, Fukuoka 812-8581, Japan; 2 Research Institute for Diseases of the Chest, Graduate School of Medical Sciences, Kyushu University, Fukuoka 812-8581, Japan; 3 Research Center for Nucleotide Pool, Kyushu University, Fukuoka 812-8581, Japan; 4 National Institute of Radiological Sciences, Chiba 263-8555, Japan; 5 Cancer Institute, Japanese Foundation for Cancer Research, Tokyo 135-8550, Japan

## Abstract

Deoxyinosine (dI) occurs in DNA either by oxidative deamination of a previously incorporated deoxyadenosine residue or by misincorporation of deoxyinosine triphosphate (dITP) from the nucleotide pool during replication. To exclude dITP from the pool, mammals possess specific hydrolysing enzymes, such as inosine triphosphatase (ITPA). Previous studies have shown that deficiency in ITPA results in cell growth suppression and DNA instability. To explore the mechanisms of these phenotypes, we analysed ITPA-deficient human and mouse cells. We found that both growth suppression and accumulation of single-strand breaks in nuclear DNA of ITPA-deficient cells depended on MLH1/PMS2. The cell growth suppression of ITPA-deficient cells also depended on p53, but not on MPG, ENDOV or MSH2. ITPA deficiency significantly increased the levels of p53 protein and *p21* mRNA/protein, a well-known target of p53, in an MLH1-dependent manner. Furthermore, MLH1 may also contribute to cell growth arrest by increasing the basal level of p53 activity.

For all organisms, maintenance of the integrity of genomic DNA and its precise transmission from cell to cell and from parents to offspring is fundamental to life. DNA, however, is susceptible to damage from various reactive molecules. Some DNA damage induces cell death or genetic mutation, and causes various disorders in humans, such as aging, cancer and hereditary diseases^1^,^2^. Base moieties of nucleic acids, which define genetic information, also suffer various chemical modifications, such as oxidation, deamination, methylation or halogenation^3^,^4^,^5^,^6^ that result in the generation of abnormal bases. These modifications can occur because of endogenous factors, such as reactive oxygen or nitrogen species, or after exposure to exogenous factors, such as ionizing radiation, ultraviolet light or chemical agents^3^,^4^,^5^,^6^. Various enzymatic reactions also generate abnormal bases in nucleic acids^7^,^8^. Direct modification of normal bases already incorporated in DNA is one of two main pathways for the accumulation of abnormal bases in DNA. The second pathway is the incorporation of abnormal deoxynucleoside triphosphates from the nucleotide pool into newly synthesized DNA during its replication. To avoid deleterious effects of the abnormal nucleotides, cells are equipped with specific enzymes to hydrolyse the abnormal nucleoside triphosphates to the corresponding monophosphates. These enzymes are known as nucleotide pool sanitizing enzymes^9^,^10^,^11^.

Deoxyinosine (dI) is an abnormal nucleoside and has hypoxanthine as its base moiety. Hypoxanthine is generated by oxidative deamination of adenine, which occurs in the presence of nitrous acid^12^, or via catalysis by specific enzymes, such as adenosine deaminase or AMP deaminase. dITP can be generated by oxidative deamination of dATP, and incorporated into DNA^10^,^13^,^14^. In addition, hypoxanthine is a base moiety of inosine monophosphate (IMP), which is a normal intermediate metabolite in the *de novo* purine nucleotide metabolism pathway. Pang *et al*. reported a large increase of dI in DNA in strains of *Escherichia coli, and Saccharomyces cerevisiae* unable to convert IMP to AMP or GMP, and unable to hydrolyze dITP/ITP^15^, suggesting the existence of a pathway from IMP, a normal nucleotide, to dI in DNA.

Previous studies in mammalian cells have revealed that inosine triphosphatase (ITPA), encoded by the *ITPA* gene, hydrolyses inosine triphosphate (ITP) and dITP to IMP and dIMP with essentially the same efficiency^16^,^17^. *Itpa* knockout (KO) mice die before weaning with features of growth retardation and heart failure^18^. These results show that ITP and dITP are produced under physiological conditions in living cells, and that they induce vital dysfunction unless hydrolysed by ITPA. Furthermore, *Itpa* KO mouse embryos had increased levels of deoxyinosine/inosine in DNA/RNA, and primary mouse embryonic fibroblasts (MEFs) derived from *Itpa* KO embryos exhibited prolonged doubling time and increased chromosome abnormalities and accumulation of single-strand breaks (SSBs) in nuclear DNA compared with primary MEFs prepared from wild-type embryos^19^.

We have previously performed a screen for ITP-binding proteins^20^ and revealed that nucleoside diphosphate linked moiety X-type motif16 (NUDT16), encoded by *NUDT16*, also hydrolyses (deoxy)inosine triphosphate and (deoxy)inosine diphosphate to (deoxy)inosine monophosphate. Knockdown of *NUDT16* in either HeLa MR cells or ITPA-deficient MEF cells causes cell cycle delay in S phase, reduced cell proliferation, and increased accumulation of SSBs in nuclear DNA, suggesting that NUDT16, along with ITPA, has an important biological function in mammals as a sanitizing enzyme against inosine nucleotides.

The human *ITPA* gene has a polymorphic variant, P32T, which has decreased enzymatic activity through three mechanisms: protein instability, decreased rate of catalysis, and improper mRNA splicing^21^,^22^,^23^. The P32T variant is associated with potentially severe adverse drug reactions towards the thiopurine drugs, azathioprine and 6-mercaptopurine^24^. Furthermore, the P32T variant is related to protection against adverse effects of Ribavirin treatment in patients with hepatitis C^25^,^26^,^27^,^28^.

It has been reported that dI generated in DNA can be excised by several DNA repair systems in prokaryotes and eukaryotes. 3-Methyl-adenine DNA glycosylase II (AlkA) in *Escherichia coli* recognizes *N*-alkylpurine adducts, deaminated purine adducts, and lipid peroxidation-induced purine adducts in DNA, and cleaves *N*-glycosylic bonds within them. AlkA excises the hypoxanthine base from dI in DNA, forms an apurine/apyrimidine site (AP site) and initiates a base excision repair (BER) reaction^29^,^30^,^31^. Mammalian N-methylpurine-DNA glycosylase (MPG), which is known to excise at least 17 structurally diverse modified bases from DNA^32^, also removes hypoxanthine from double-stranded DNA (dsDNA), thus initiating BER^33^,^34^. Endonuclease V, a product of the *nfi* gene of *E. coli*, recognizes dI in DNA and cleaves the DNA at the second phosphodiester bond 3′ to the dI, leaving a nick and initiates the alternative excision repair (AER) system^35^,^36^,^37^. It has been reported that human and mouse also have endonuclease V homologs^38^,^39^. Mi *et al*. have reported that human endonuclease V (hENDOV) recognizes and repairs dI-containing dsDNA, according to the following order of dI pairings: dI in single-stranded DNA > dG:dI > dT:dI > dA:dI > dC:dI^38^. On the other hand, two other groups independently reported that recombinant hENDOV prefers inosine-containing single-stranded RNA but not dI-containing dsDNA as its substrate^40^,^41^,^42^, so the function of hENDOV against dI in DNA is currently controversial.

The DNA mismatch repair system (MMR) plays an important role in maintaining genome stability by correcting both base-base mismatches and insertion/deletion mispairs generated during DNA replication^43^. In the process of MMR, the newly synthesized DNA strand is incised and a DNA fragment longer than 150 bases and containing the incorrect base is removed. Defects in MMR increase the spontaneous mutation rate. Moreover, it has been established that the MMR system is required for cell cycle arrest and/or programmed cell death in response to certain types of DNA damage^44^,^45^,^46^.

In the present study, to understand the mechanism that causes cell growth delay and SSBs in nuclear DNA in ITPA-deficient cells, we analysed ITPA-deficient human and mouse cells, and revealed that ITPA deficiency induces MLH1/PMS2- and p53-dependent growth arrest and DNA instability in mammalian cells.

## Results

### MLH1 is involved in cell growth delay caused by *NUDT16* knockdown in HeLa MR cells

We previously reported that knockdown of *NUDT16* in HeLa MR cells, which are derived from human cervical cancer cells, caused growth delay^20^. We performed triple knockdown of *NUDT16, MPG* and *ENDOV* in HeLa MR cells to confirm whether these repair enzymes (MPG and ENDOV) are involved in the cell growth delay induced by knockdown of *NUDT16* because bacterial endonuclease V was reported to cause DNA instability if dITP accumulates in bacterial cells^13^. Although single knockdown of *MPG* or *ENDOV* did not affect cell growth of HeLa MR cells, double knockdown of *MPG* and *ENDOV* induced significant cell growth delay similar to that seen for single knockdown of *NUDT16* (Supplementary Figures S1 and S2). Knockdown of *MPG* and *ENDOV* in addition to *NUDT16* knockdown (KD) did not rescue the phenotype of NUDT16 deficiency but caused additional cell growth delay. From these results, we suspected that other DNA repair enzymes might be involved in the growth delay caused by *NUDT16* KD. We considered MLH1 as such a candidate because dI has been reported to pair with any normal deoxynucleotide in DNA, although its stability depends on opposite bases^47^. Double knockdown of *MLH1* and *NUDT16* rescued the cell growth delay induced by single knockdown of *NUDT16*. We also analysed the influence of *ITPA* KD; however, this did not affect HeLa MR cell growth (Supplementary Figure S1). These results suggest that the MMR pathway, but not MPG or ENDOV, is involved in the cell growth delay induced by accumulation of dITP or ITP caused by knockdown of *NUDT16*.

### ITPA deficiency induces cell growth delay in normal human cells

**Figure 1:**
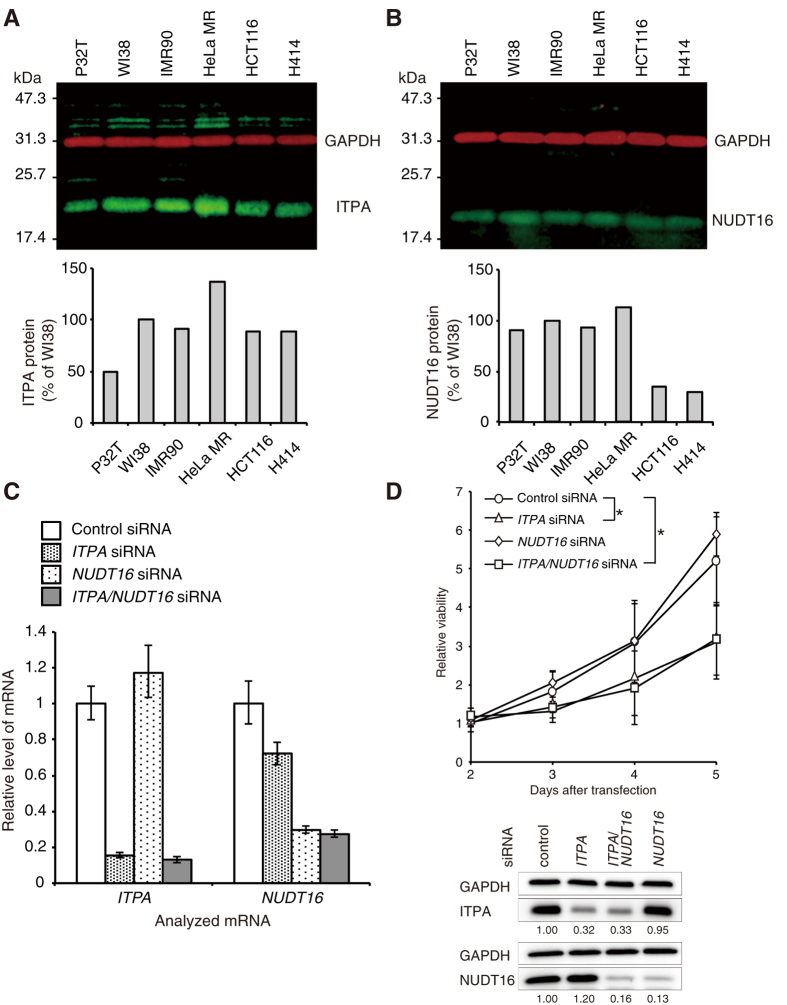
Knockdown of *ITPA* induces cell growth delay in WI38 cells. (**A**) Western blot analysis of ITPA in human cell lines. Whole cell lysates were analysed with anti-ITPA and anti-GAPDH antibodies. The primary antibodies were detected with fluorophore-conjugated secondary antibodies. ITPA (green) and GAPDH (red) signals were detected and quantified using the Odyssey system. The bar graph shows relative levels of ITPA normalized to those of GAPDH. (**B**) Western blot analysis of NUDT16 in human cell lines. NUDT16 (green) and GAPDH (red) were detected in whole cell extracts as described for panel a. Normalized NUDT16 levels are shown in the bar graph. (**C**) Levels of *ITPA* and *NUDT16* mRNAs in WI38 cells transfected with control, *ITPA* and/or *NUDT16* siRNA. Two days after siRNA transfection, cells were subjected to analysis for RNA levels by real time quantitative RT-PCR. Data for each mRNA were normalized relative to that for 18S rRNA. Levels relative to control cells transfected with control siRNA are shown as the mean ± SD (n = 3). (**D**) Viability of WI38 cells transfected with control, *ITPA* and/or *NUDT16* siRNA. Cell viability was measured using the Cell Counting Kit-8, 2, 3, 4, and 5 days after transfection. ITPA and NUDT16 protein levels 3 days after the siRNA transfections were confirmed by western blot analysis with antibodies against ITPA, NUDT16 and GAPDH. Relative protein levels normalized to GAPDH are shown under the blot images. Results are tested with two-way ANOVA, *P* = 0.0002; Tukey’s HSD *post hoc* test, *P* = 0.0256 (control versus *ITPA* siRNA), *P* = 0.0179 (control versus *ITPA* and *NUDT16* siRNA). Data are presented as the mean ± SD (n = 3).

We previously reported that ITPA-deficient primary MEFs show growth delay compared with wild-type MEFs^19^. It is known that p53 and Rb proteins are inactivated by E6 and E7 gene products produced by human papilloma virus genes integrated in the HeLa genome^48^,^49^. As a result, the DNA damage response is not normal in HeLa cells. Therefore, we next used WI38 fibroblast cells, derived from human normal embryonic lung, to analyse ITPA deficiency in normal human cells. We analysed the ITPA cDNA sequence from WI38 cells and confirmed that WI38 expresses mRNA encoding wild-type ITPA protein (accession no. NP_258412.1). Western blot analysis showed that the level of ITPA in WI38 cells was essentially the same as in various other cell lines, including HeLa MR cells, another normal fibroblast cell, IMR90, and cell lines derived from human colorectal cancer (HCT116 and H414). The one exception was GM01617 (P32T) cells, a human fibroblast cell line that encodes the unstable ITPA P32T variant from both alleles (Fig. 1A)^50^. The level of NUDT16 protein in WI38 cells was also similar to that in HeLa MR cells and higher than that in human colorectal cancer cell lines (Fig. 1B). We performed knockdown experiments with the WI38 cell line, and transfection of siRNA for *ITPA* or *NUDT16* efficiently decreased the expression of both genes (Fig. 1C). After transfection of control, *ITPA* or *NUDT16* siRNA, proliferation of WI38 cells was analysed using a colorimetric assay. *ITPA* KD, but not *NUDT16* KD, induced significant cell growth delay compared with control (Fig. 1D, upper panel). Whole cell extracts prepared from WI38 cells 4 days after siRNA transfection were subjected to western blot analysis of ITPA and NUDT16 proteins. This confirmed the efficient suppression of ITPA and/or NUDT16 expression (Fig. 1D, lower panels). These results reveal that ITPA deficiency induces cell growth delay in normal human cells as well as in MEF cells.

### Cell growth delay caused by ITPA deficiency depends on MLH1

To confirm more precisely the contribution of MLH1 to the cell growth delay induced by ITPA deficiency, we performed *ITPA* KD in MLH1-deficient HCT116 cells, which are derived from human colorectal cancer cells, and MLH1-proficient H414 cells, which are derived from HCT116 cells as described in Supplementary Information (Figure S3). The HCT116 cell line is homozygous for a point mutation that changes serine (TCA) to stop (TAA) at amino acid residue 252 in exon 9 of the *MLH1* gene located on chromosome 3, and thus this cell line is completely deficient in MLH1 protein^51^. HCT116 has wild-type p53, a near diploid karyotype (n = 45) and stable chromosome number^52^. HCT116 also lacks normal MSH3 expression^53^. We have established a cell line expressing wild-type MLH1 protein by targeted knock-in of the wild-type *MLH1* coding sequence into one allele of the parental HCT116 cell line and named the line H414.

**Figure 2:**
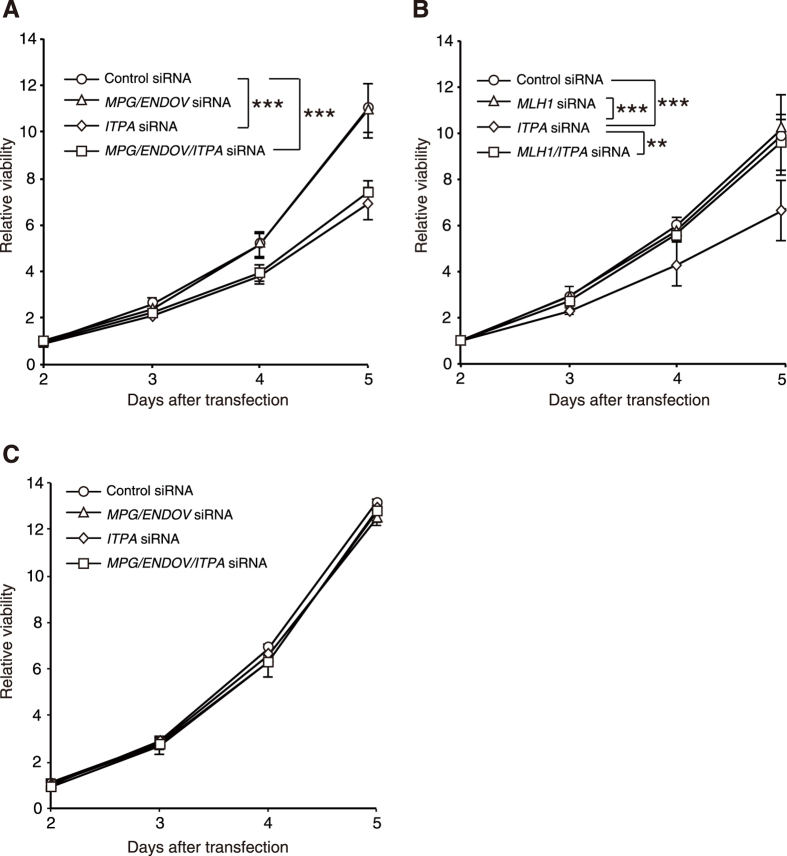
MLH1 is necessary for cell growth delay caused by *ITPA* knockdown in H414 cells. (**A**) H414 cells were transfected with *ITPA, MPG* and/or *ENDOV* siRNA. Results are tested with two-way ANOVA, *P* < 0.0001; Tukey’s HSD *post hoc* test, *P* < 0.001 (control versus *ITPA* siRNA or control versus *MPG*/*ENDOV*/*ITPA* siRNA). (**B**) H414 cells were transfected with *ITPA* and/or *MLH1* siRNA. Results are tested with two-way ANOVA, *P* < 0.0001; Tukey’s HSD *post hoc* test, *P* < 0.001 (control versus *ITPA* siRNA or *MLH1* versus *ITPA* siRNA), *P* = 0.0014 (*ITPA* versus *ITPA*/*MLH1* siRNA). (**C**) HCT116 cells were transfected with *ITPA, MPG* and/or *ENDOV* siRNA. Results tested with two-way ANOVA showed no significance. Cell viability was analysed using the Cell Counting Kit-8. Data are presented as the mean ± SD (n = 3).

We transfected siRNAs for various genes into HCT116 and H414 cells and analysed their cell growth rates. Knockdown of *ITPA* induced significant cell growth delay in H414 cells. Triple knockdown of *ITPA, MPG* and *ENDOV* also caused growth delay in H414 cells, but the delay was essentially the same extent as that caused by knockdown of *ITPA* alone (Fig. 2A and S4A). Double knockdown of *ITPA* and *MLH1* did not induce any change in cell growth, suggesting that MLH1 knockdown completely cancelled the cell growth delay induced by single knockdown of *ITPA* (Fig. 2B and S4B). On the other hand, in HCT116 cells, *ITPA* KD had no influence on their growth. Moreover, triple knockdown of *ITPA, MPG* and *ENDOV* had no effect on the proliferation of HCT116 cells (Fig. 2C and S4C). These results revealed that the cell growth delay caused by ITPA deficiency exclusively depends on MLH1 function, while MPG and ENDOV are not involved.

### Nuclear DNA instability induced by ITPA deficiency also depends on MLH1

**Figure 3:**
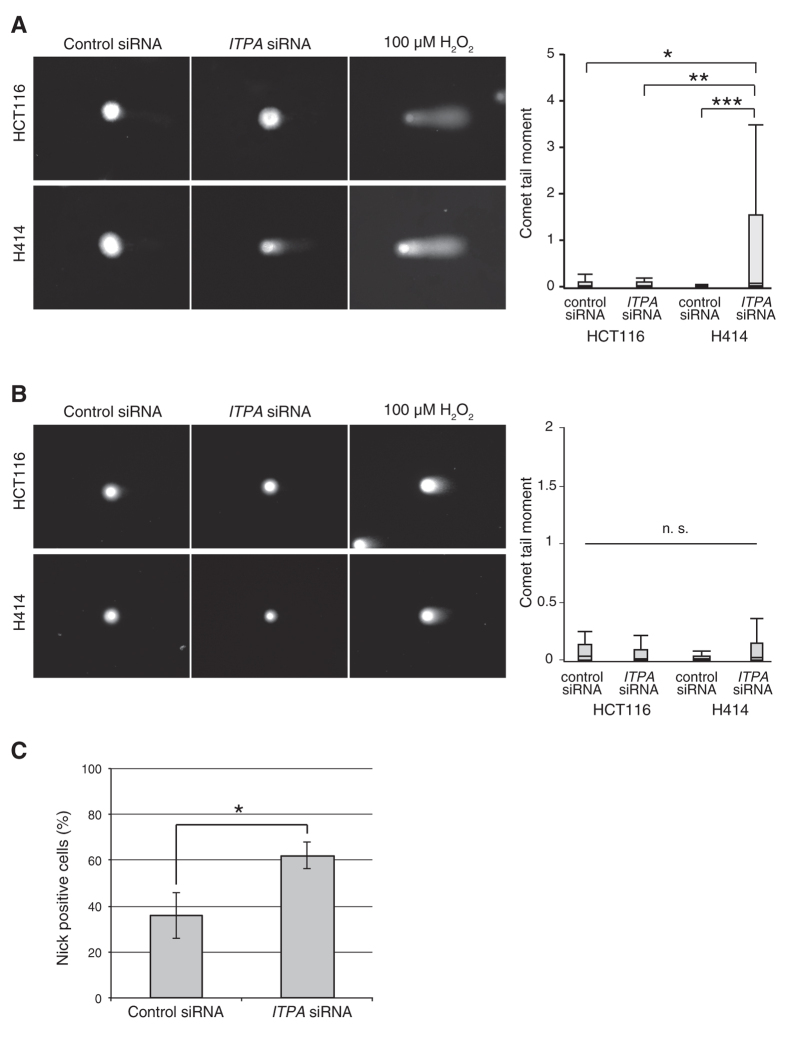
MLH1 deficiency reduces accumulation of single-strand breaks in nuclear DNA caused by *ITPA* knockdown in H414 cells. Cells were analysed using the comet assay 4 days after transfection with siRNAs. (**A**) Comet assay under alkaline conditions. Tail moments of at least 30 cells were calculated for each group. Comet images and calculated tail moments are shown in panels and box-and-whisker plots, respectively. Results tested with the Steel-Dwass test, *P* < 0.001 (control siRNA in H414 versus *ITPA* siRNA in H414), *P* = 0.0255 (control in HCT116 siRNA versus *ITPA* siRNA in H414), *P* = 0.0011 (*ITPA* siRNA in HCT116 versus *ITPA* siRNA in H414). (**B**) Comet assay under neutral conditions. Tail moments were not significantly increased in either group. The assays were independently performed three times. (**C**) *In situ* nick translation. The numbers of cells with or without fluorescein signal indicating nicks were counted. The mean ± SD (n = 3) of the ratio of nick-positive cells in each group is shown. Data were tested with Student’s t-test, *P* = 0.0169.

We previously reported that *Itpa*-deficient primary MEFs accumulate SSBs in nuclear DNA and exhibit growth delay^19^. Therefore, we performed comet assays with HCT116 and H414 cells after *ITPA* KD to analyse the accumulation of SSBs in nuclear DNA. In alkaline conditions, H414 cells transfected with *ITPA* siRNA showed significantly increased comet tails and tail moments compared with cells transfected with control siRNA (Fig. 3A). In neutral conditions, there was no significant difference in comet tail moments between the two groups of cells (Fig. 3B). These results indicate that ITPA deficiency induced accumulation of SSBs or alkaline-labile sites such as abasic sites which are converted to SSBs in alkaline conditions, in the nuclear DNA of H414 cells. On the other hand, HCT116 cells transfected with *ITPA* siRNA did not exhibit any significant increase in comet tail moments in alkaline or neutral conditions compared with control HCT116 cells. These results suggest that ITPA deficiency induces accumulation of SSBs or alkaline-labile sites in nuclear DNA through an MLH1-dependent pathway. To analyze nuclear genome instability using a different assay system, we performed an *in situ* nick translation assay at neutral pH with H414 cells three days after siRNA transfection as described in Supplemental Materials and Methods, because the alkaline comet assay may detect alkaline-labile sites such as abasic sites. H414 cells were transfected independently three times for each siRNA. The cells with nick-dependent signals were significantly increased by *ITPA* KD from 35.9% in control to 62.0%, as shown in Fig. 3C. This result suggests that the increased tail moment following *ITPA* KD in Fig. 3A results from accumulated SSBs in nuclear genomic DNA.

### Deoxyinosine in the culture medium induces cell growth delay in H414 cells

**Figure 4:**
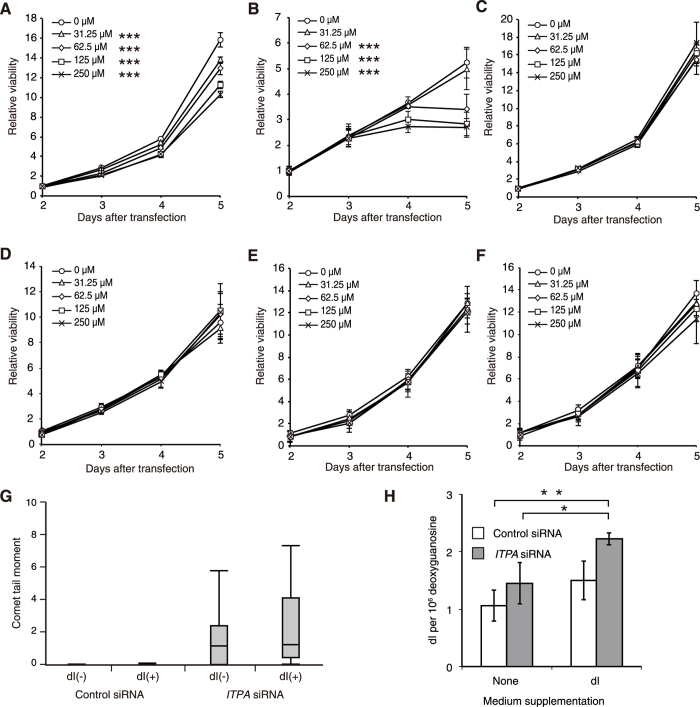
Deoxyinosine in the culture medium induces concentration-dependent cell growth delay in H414 cells. Cells were transfected with siRNAs, and the culture medium was supplemented with deoxyinosine (dI) or inosine (rI) 24 h after transfection. Cell viability was measured using the Cell Counting Kit-8, 1, 2, 3, and 4 days after the supplementation. (**A**) H414 cells transfected with control siRNA were treated with the indicated concentration of deoxyinosine. Results tested with two-way ANOVA, *P* < 0.001; Tukey’s HSD *post hoc* test, *P* < 0.001, compared with control. (**B**) H414 cells transfected with *ITPA* siRNA were treated with dI. Results tested with two-way ANOVA, p < 0.001; Tukey’s HSD *post hoc* test, *p* < 0.001, compared with control. (**C**) H414 cells transfected with control siRNA were treated with rI. Results tested with two-way ANOVA shows no significance. (**D**) H414 cells transfected with *ITPA* siRNA were treated with rI. Results tested with two-way ANOVA shows no significance. (**E**) HCT116 cells transfected with control siRNA were treated with dI. Results tested with two-way ANOVA show no significance. (**F**) HCT116 cells transfected with *ITPA* siRNA were treated with dI. Results tested with two-way ANOVA show no significance. Data are presented as the mean ± SD (n = 3). (**G**) H414 cells cultured with 250 μM of dI for three days were analyzed by the comet assay under alkaline conditions. Tail moments of at least 18 cells were calculated for each group. The calculated tail moments are shown in box-and-whisker plots. The results were analysed with the Steel-Dwass test, and no significant increase in tail moments was observed between *ITPA*-KD cells with dI and *ITPA*-KD cells without dI. (**H**) DNA samples were prepared from H414 cells 4 days after 250 μM dI supplementaion, and their dI contents were analysed by LC–MS/MS. Results tested with two-way ANOVA, *P* = 0.0096 for *ITPA*-KD treatment, and *P* = 0.0066 for dI treatment. Tukey’s HSD *post hoc* test, *P* = 0.0048 (Control/none versus *ITPA*/dI), *P* = 0.0442 (*ITPA*/none versus *ITPA*/dI), and *P* = 0.0572 (Control/dI versus *ITPA*/dI). Data are presented as the mean ± SD (n = 3).

The data described above indicated that the dITP accumulated in ITPA-deficient cells might be incorporated into nuclear DNA during its replication, and that SSBs in nuclear DNA were produced during the process of MMR, resulting in cell growth delay. To confirm whether intracellular dITP can cause cell growth delay, we analysed the effects of dI added into the culture medium. It is known that nucleosides in culture media can diffuse or be efficiently imported into cells by nucleoside transport proteins and can then be phosphorylated to nucleoside triphosphate by several kinases^54^,^55^,^56^. Addition of dI into the culture medium induced growth delay in H414 cells transfected with control siRNA in a dose-dependent manner (Fig. 4A), while H414 cells transfected with *ITPA* siRNA showed more severe delay in growth after addition of dI (Fig. 4B). On the other hand, addition of inosine did not change the proliferation rate of H414 cells transfected with control or *ITPA* siRNA (Fig. 4C,D). In HCT116 cells, there was no change in their proliferation rate after dI addition (Fig. 4E,F). These results suggest that dITP but not ITP induces cell growth delay in an MLH1-dependent manner. To clarify whether deoxyinosine nucleotides increase the single strand breakage of DNA, we subjected H414 *ITPA*-KD cells cultured with 250 μM dI to the comet assay (Fig. 4G). Overall comet tail moments were not significantly different between the dI-treated cells (dI+) and the control cells (dI−) after *ITPA* KD. However, 25^th^ percentile values were 0.00215 in the control group and 0.45612 in the dI-treated group. These values are 0.29% and 38.12% of the 50^th^ percentile value in each group respectively. Thus, dI treatment of ITPA-deficient cells tended to increase the ratio of cells with considerable tail moment. We cannot deny the possibility that supplementation of dI into the culture medium might disrupt nucleotide metabolism, thereby resulting in the incorporation of other nucleotide intermediates into the DNA or causing dNTP pool imbalances. Thus, we quantitatively measured dI content in genomic DNA of H414 cells after *ITPA* KD and dI supplementation of the medium (Fig. 4H). Two-way ANOVA analysis of the results revealed that each *ITPA* KD (*P* = 0.0096) and dI treatment (*P* = 0.0066) is an independent factor that significantly changes dI content in the DNA. Tukey’s HSD post hoc test among all groups showed that dI content is the highest in *ITPA*-KD/dI-treatment group with significant difference compared to those in *ITPA* KD without dI treatment (*P* = 0.0442) or control siRNA without dI treatment (*P* = 0.0048). These results indicate that the cellular responses induced by dI treatment, especially under ITPA deficiency, can be attributed to the incorporation of dI into the DNA, even if dI might disrupt nucleotide metabolism. Considering the contribution of dI incorporation into the DNA to its biological consequence, we noticed that majority of the basal dI content in H414 cells without *ITPA* KD or dI treatment may reflect dI generated by deamination of deoxyadenosine in the DNA during cell culture, because H414 cells without *ITPA* KD nor dI treatment have normal level of ITPA without dI supplementation, thereby avoiding dI incorporation into the DNA. On the other hand, the increase in dI content from the basal level after *ITPA* KD and/or dI treatment probably represent net incorporation of dI into DNA during DNA synthesis, and that the dI incorprated into the DNA, not the basal dI generated by deamination, may have caused growth arrest dependent on MLH1.

### MLH1 deficiency partially rescues cell growth delay in ITPA-deficient primary MEFs but has no influence on the accumulation of deoxyinosine in nuclear DNA

**Figure 5:**
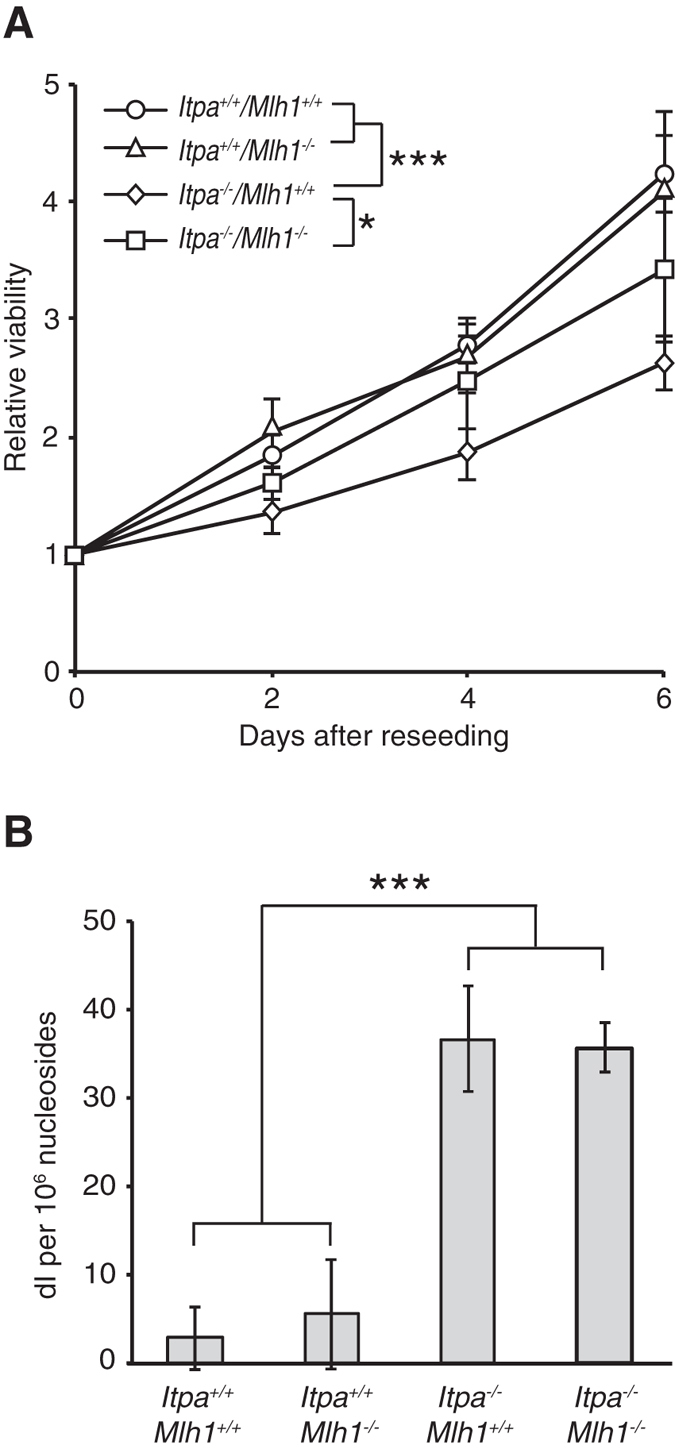
MLH1 deficiency partially rescues cell growth delay in ITPA-deficient primary mouse embryonic fibroblast (pMEF) cells, but had no effect on the accumulation of deoxyinosine in nuclear DNA of mouse embryos. (**A**) Viability of pMEF cells. For each genotype, pMEF cells were independently prepared from at least five or six E14.5 embryos obtained from intercrossing double heterozygous mice (*Itpa*^+/−^/*Mlh1*^+/−^). Cell viability was measured using the Cell Counting Kit-8, 0, 2, 4 or 6 days after reseeding. Results tested with two-way ANOVA, *P* < 0.0001; Tukey’s HSD *post hoc test, P* < 0.0001 (*Itpa*^+/+^/*Mlh1*^+/+^ versus *Itpa*^−/−^/*Mlh1*^+/+^, *Itpa*^+/+^/*Mlh1*^−/−^ versus *Itpa*^−/−^/*Mlh1*^+/+^), *P* =0.036 (*Itpa*^−/−^/*Mlh1*^+/+^ versus *Itpa*^−/−^/*Mlh1*^−/−^). (**B**) Deoxyinosine levels in nuclear DNA of mouse embryos. DNA samples were independently prepared from the heads of five E14.5 embryos for each genotype, and their dI contents were analysed by LC–MS/MS. Results tested with one-way ANOVA, *P* < 0.001. Tukey’s HSD *post hoc* test, *P* < 0.001 (*Itpa*^+/+^/*Mlh1*^+/+^ or *Itpa*^+/+^/*Mlh1*^−/−^ versus *Itpa*^−/−^/*Mlh1*^+/+^ or *Itpa*^−/−^/*Mlh1*^−/−^). Data are presented as the mean ± SD (n = 5 or 6).

To examine ITPA deficiency-induced cell growth delay and its dependency on MLH1 in primary MEFs, we analysed proliferation rate of primary MEFs prepared from embryos derived from intercrosses of double heterozygous mice (*Itpa*^+/−^/*Mlh1*^+/−^), as described in the Supplementary Information. As in our previous report, *Itpa*-KO MEFs (*Itpa*^−/−^/*Mlh1*^+/+^) showed significant growth delay compared with wild-type MEFs (*Itpa*^+/+^/*Mlh1*^+/+^)^19^. Double KO MEFs (*Itpa*^−/−^/*Mlh1*^−/−^) also showed a delay in growth; however, the delay was significantly less than that in *Itpa*-single-KO MEFs (*Itpa*^−/−^/*Mlh1*^+/+^) (Fig. 5A). These results indicate that the growth delay in ITPA-deficient primary MEFs also depends, in part, on MLH1.

Next, we examined the accumulation of dI residues in nuclear DNA prepared from ITPA-and/or MLH1-deficient mouse embryos by liquid chromatography coupled with tandem mass spectrometry (LC–MS/MS). These analyses revealed that *Itpa*-KO embryos (*Itpa*^−/−^/*Mlh1*^+/+^) contained a significantly increased amount of dI in their nuclear DNA compared with wild-type embryos (*Itpa*^+/+^/*Mlh1*^+/+^). Furthermore, double KO embryos (*Itpa*^−/−^/*Mlh1*^−/−^) contained an increased amount of dI, similar to *Itpa*^−/−^ and *Mlh1*^+/+^ embryos (Fig. 5B). In addition, we analysed the genotype of 74 mice obtained from intercrossing double heterozygous mice (*Itpa*^+/−^/*Mlh1*^+/−^). As shown in Supplementary Table S1, no *Itpa*^−/−^ mice were detected among these offspring despite Mendel’s law predicting the identification of approximately 18 ITPA-deficient mice. From these data, we concluded that MLH1 deficiency can rescue the growth delay of ITPA-deficient mouse cells, but cannot rescue the perinatal lethality of ITPA-deficient mice.

### ITPA deficiency induces G1 arrest in H414 cells

**Figure 6:**
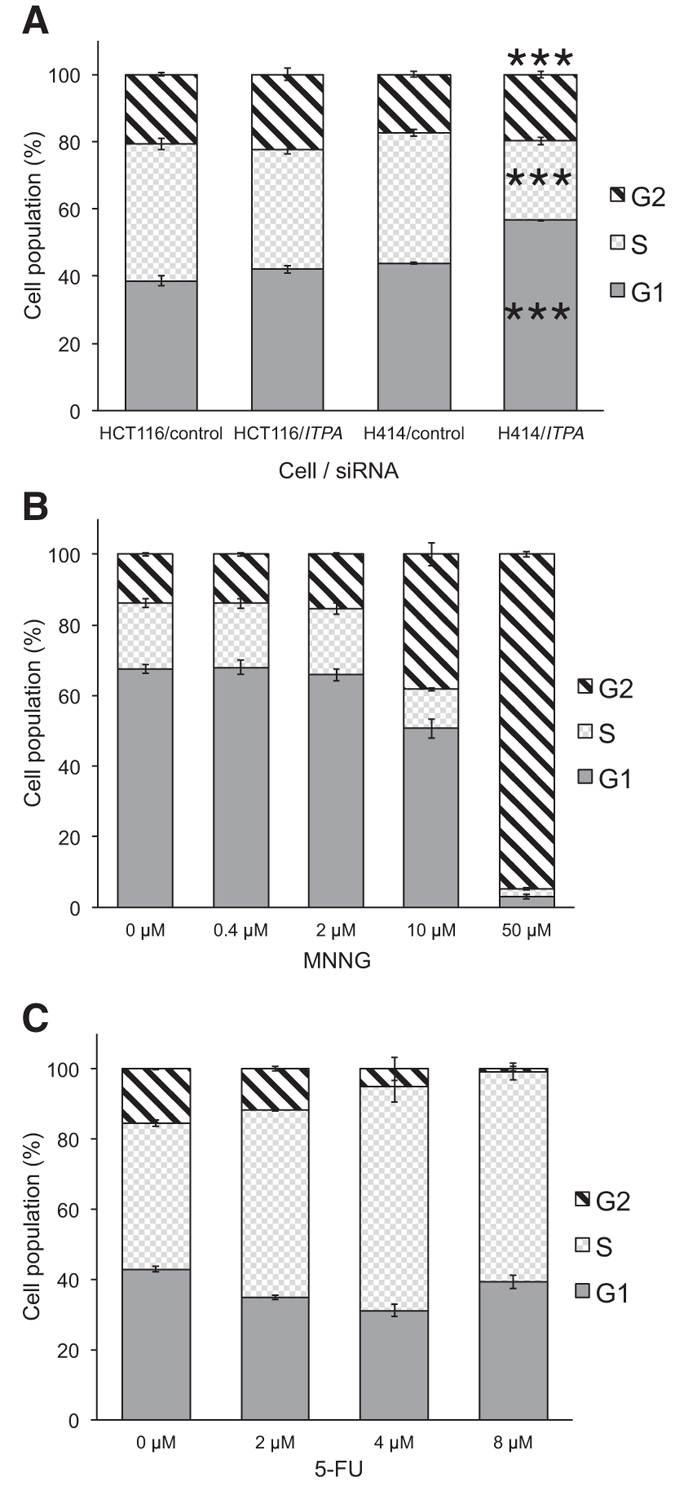
ITPA deficiency but not 5-FU or MNNG induced G1 cell cycle arrest. Cell cycle analysis was performed using flowcytometry and ModFitLT software. (**A**) H414 and HCT116 cells were harvested three days after *ITPA* KD (n = 4). (**B**) H414 cells were treated with MNNG for 1 h, and then cultured without MNNG for 3 more days before harvest (n = 3). (**C**) H414 cells were cultured in the presence of 5-FU for 24 h and then harvested (n = 3). The mean ± SD is shown for G1, S, and G2 phases for each group. Results were tested with Pearson’s chi-square test or Fisher’s exact test in (**A**), *P* < 0.001 (total H414 cells with *ITPA* siRNA versus others, H414 cells with *ITPA* siRNA versus others in G1, and H414 cells with *ITPA* siRNA versus others in S phase).

We analyzed the cell cycle of H414 and HCT116 cells 3 days after *ITPA* KD by flow cytometric analysis. *ITPA* KD significantly decreased the number of cells in S phase and increased the number of cells in G1 phase compared with negative control cells in H414 but not HCT116 cells (Fig. 6A), indicating G1 cell cycle arrest. To compare *ITPA* KD with other agents that induce cell cycle arrest, we treated H414 cells with N-methyl-N′-nitro-N-nitrosoguanidine (MNNG) or fluorouracil (5-FU) and analyzed their cell cycles (Fig. 6B,C). MNNG induces cell cycle arrest through the MMR-mediated futile cycle^57^. The treatment of H414 cells with MNNG for 1 h induced accumulation of cells in G2 phase 72 h after the treatment in a dose-dependent manner, and treatment with 5-FU for 48 h caused S phase arrest. Thus, G1 arrest induced by ITPA deficiency is different from G2 or S phase cell cycle arrest induced by MNNG or 5-FU.

### PMS2 is necessary for cell growth suppression induced by ITPA deficiency, but MSH2 is dispensable

**Figure 7:**
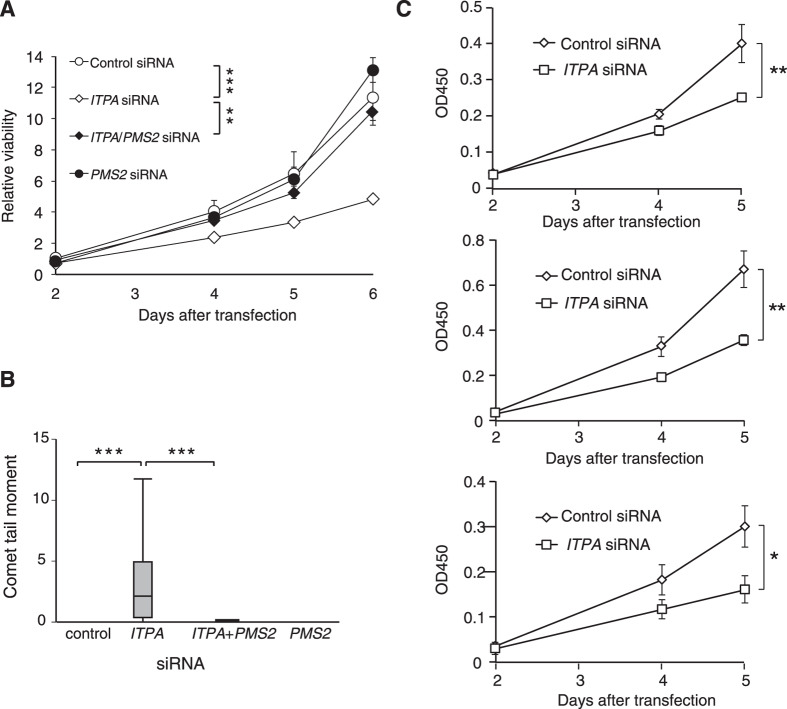
PMS2, but not MSH2, contributes to cell growth arrest and DNA instability caused by *ITPA* knockdown. H414 cells were transfected with *ITPA* and/or *PMS2* siRNA, and then analysed using the Cell Counting Kit-8 (**A**) and comet assay in alkaline conditions (**B**) to assess cell viability and DNA damage, as in Figs 2 and 3A, respectively. For the comet assay, tail moments of at least 16 cells were calculated for each group. (**C**) Three H414-MSH2 deficient clones (#3–52 in upper, #3–113 in middle, and #3–119 in lower graph) were transfected with *ITPA* or negative control siRNA. Cell viability was analysed using the Cell Counting Kit-8. Data are presented as the mean ± SD (n = 3) in (**A**,**C**), or as box-and-whisker plots in (**B**). Results were tested with two-way ANOVA, *P* < 0.001; and Tukey’s HSD *post hoc* test (**A**), with two-way ANOVA, (control versus *ITPA* siRNA for each line) (**C**), or with the Steel-Dwass test, *P* < 0.0001 (control siRNA versus *ITPA* siRNA), *P* < 0.0001 (*ITPA* siRNA versus *ITPA* plus *PMS2* siRNA) (**B**).

To evaluate contributions of other components of the MMR system, we examined effects of PMS2 or MSH2 deficiency in H414 cells. PMS2 forms a heterodimer with MLH1, and MSH2, together with MSH6 or MSH3, is essential for recognition of base mismatches in DNA. First, we performed knockdown of *ITPA* and *PMS2* in H414 cells and analyzed cell growth and nuclear DNA damage with a Cell Counting Kit-8 and a comet assay respectively. Knockdown of *PMS2* nullified both growth suppression and DNA damage in ITPA-deficient H414 cells (Fig. 7A,B). For MSH2, we established three MSH2-deficient H414 clones (#3–52, #3–113, and #3–119) using CRISPR/Cas9 technology. Each of the clones has a base insertion and/or a base deletion mutation on both *MSH2* alleles as summarized in Supplementary Table S2. The mutations caused frame shifts and termination of translation by a new stop codon in exon 4 of each *MSH2* allele. Western blot analysis confirmed MSH2 deficiency in these cell lines (Supplementary Figure S5A). Among the three clones, #3–113 proved to be resistant to 6-thioguanine, similar to MLH1-deficient HCT116 cells, thus clone #3–113 was deficient in MSH2 function (Supplementary Figure S5B). Each of these MSH2-deficient H414 clones showed significant suppression of cell proliferation after *ITPA* KD, similar to that in H414 cells with wild-type MSH2, indicating that the growth suppression caused by *ITPA* KD is independent of MSH2 (Fig. 7C).

### ITPA deficiency induces p53 and p21

**Figure 8:**
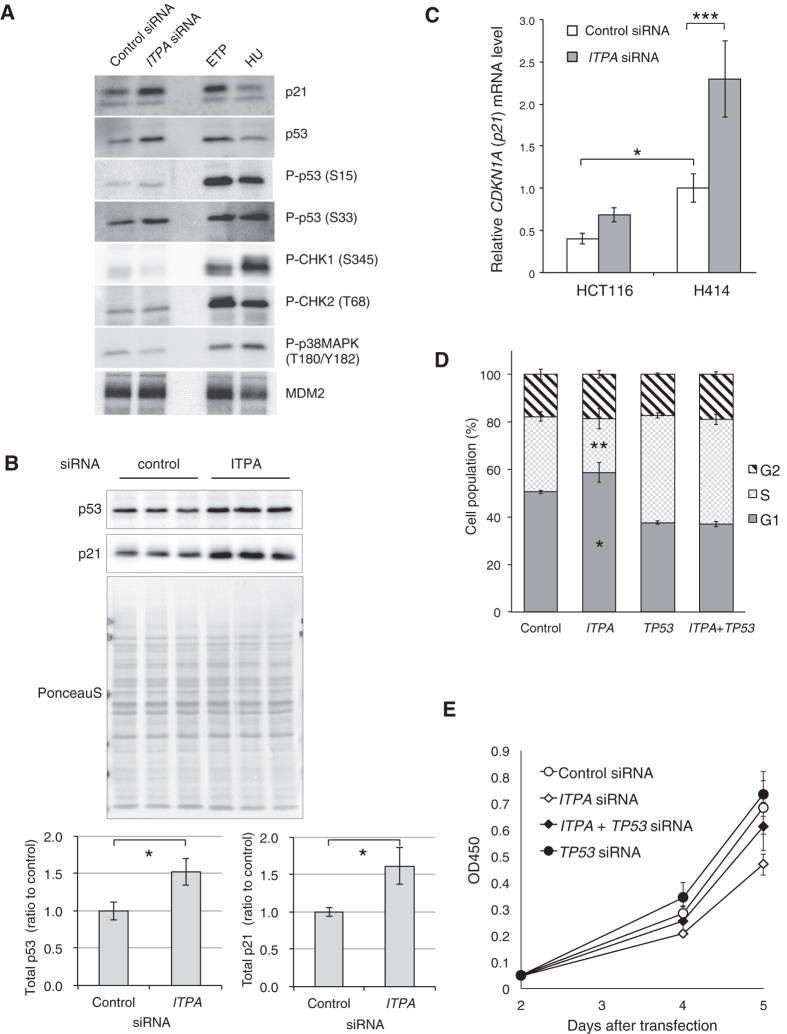
ITPA knockdown up-regulates p53 protein and p21 mRNA/protein. H414 and/or HCT116 cells were transfected with siRNAs. The cells were reseeded 24h after the transfection, and cultured further. (**A**) Three days after the transfection of control or *ITPA* siRNA, the H414 cells were analysed by western blotting with antibodies against indicated antigens. H414 cells treated with 20 μM etoposide for 8 h (ETP), or with 10 mM hydroxyurea for 2 h (HU) were used as positive control samples. (**B**) H414 cells were transfected with control or *ITPA* siRNA independently three times. After culture for 4 days, cells were harvested and whole cell extracts were subjected to western blot analysis with antibodies to p53 and p21. Blots were stained with Ponceau S to visualize total protein. The signals for p53, p21, and total protein are shown in the upper panels. The p53 or p21 levels normalized to Ponceau S staining are shown as the mean ± SD (n = 3) in the bar graphs of the lower panel. (**C**) Three days after the transfection of control or *ITPA* siRNA, HCT116 and H414 cells were harvested for an analysis of *CDKN1A (p21*) mRNA by real time RT-PCR. (**D**) Three days after the transfection of control, *ITPA*, and/or *TP53* siRNA, the cell cycle of H414 cells was analysed. (**E**) At the indicated time points after the transfection of control, *ITPA*, and/or *TP53* siRNA, H414 cells were analysed for cell viability. Results were tested with Student’s t-test, *P* = 0.0142 for p53, and *P* = 0.0133 for p21 (**B**), with two-way ANOVA and Tukey’s HSD *post hoc* test, *P* < 0.001 (H414 with control siRNA versus H414 with *ITPA* siRNA), *P* = 0.0226 (HCT116 with control siRNA versus H414 with control siRNA) (**C**,**E**) or with Fisher’s exact test, *P* = 0.0315 (H414 with control siRNA versus H414 with *ITPA* siRNA in G1), *P* = 0.0073 (H414 with control siRNA versus H414 with *ITPA* siRNA in S) (**D**).

To analyze the status of proteins related to cell cycle arrest, we harvested H414 cells 3 days after transfection of *ITPA* siRNA. Whole cell extracts were then subjected to western blot analysis. Levels of total p21 and p53 were increased after *ITPA* KD, but no induction of phosphorylation of CHK1, CHK2, or p38MAPK was observed (Fig. 8A). To analyze the induction of p53 and p21 proteins quantitatively, we repeated *ITPA* KD in H414 cells independently three times. Whole cell extracts were prepared 4 days later, and subjected to western blot analysis of p53 or p21 proteins (Fig. 8B). ITPA deficiency significantly increased both p53 and p21 protein levels. *ITPA* KD induced a mild increase of p53 protein phosphorylated at Ser15 or Ser33. To analyze *p21* mRNA, H414 and HCT116 cells were harvested 3 days after transfection of *ITPA* siRNA, and extracted mRNA subjected to real time RT-PCR. The basal level of *p21* mRNA in H414 cells was higher than that in HCT116 cells (*p* = 0.0226) (Fig. 8C). *ITPA* KD caused a significant increase in the level of *p21* mRNA in H414 but not in HCT116 cells (*p* < 0.001). To address the contribution of p53, we performed *p53* knockdown together with *ITPA* KD in H414 cells, and analyzed their cell cycle and cell proliferation using flow cytometry and the Cell Counting Kit-8 assay, respectively. *ITPA* KD alone caused a significant increase in the number of cells in G1 phase and a decrease in the number of cells in S phase, but did not cause any change in the cell cycle when *p53* was also knocked down (Fig. 8D). *p53* KD reduced the suppression of cell growth by *ITPA* KD although the effect was not significant (*p* = 0.1922) (Fig. 8E). *p53* KD induced a reduction in the level of *p21* mRNA, indicating that the elevated basal *p21* mRNA level in H414 depends on p53 (Supplementary Figure S4D). *ITPA* KD did not increase *p53* mRNA levels. Thus, the increase of p53 protein seems to depend on its stabilization.

## Discussion

**Figure 9:**
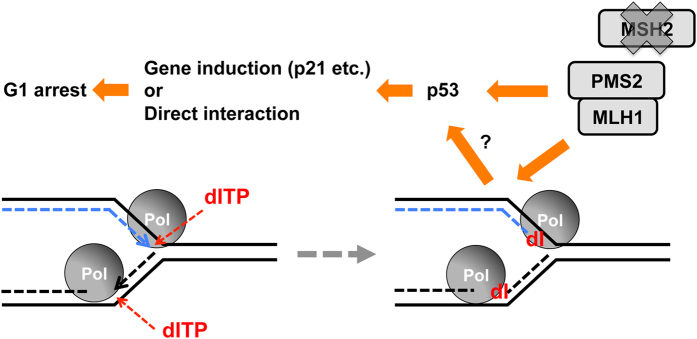
Proposed mechanism of G1 cell cycle arrest induced by dITP. In the absence of ITPA, accumulated dITP in the nucleotide pool is utilized as a substrate for DNA synthesis by DNA polymerase, thus stalling the polymerase. The stalled polymerase or deoxyinosine incorporated into DNA triggers MLH1/PMS2-dependent and MSH2-independent formation of SSBs in DNA. Accumulation of SSBs in DNA or other events caused by MLH1/PMS2 may induce G1 cell cycle arrest via stabilization of p53 protein. The p53 can induce its transcriptional targets including *CDKN1A (p21*) or affect other targets by direct interaction. MLH1 also increases the basal activity of p53.

Last year, an early-infantile encephalopathy caused by *ITPA* mutations was reported^58^. The patients show encephalopathy, progressive microcephaly, seizures, variable cardiac defect, and early death. Most patients were small for the gestational age at birth. Recently, Nakauchi A *et al*. reported polymorphic variants of *ITPA*, including a P32T variant, as susceptibility genes for young-onset tuberculosis, which may result in immune system deficiency^59^. To develop treatments for these disorders it is crucial to understand the molecular mechanisms of the phenotypes caused by ITPA deficiency. ITPA deficiency may affect cells through multiple mechanisms, including competition between ATP and accumulated ITP, and alteration of RNA/DNA by incorporation of (d)ITP. In this paper, we focused on growth arrest and genome instability of proliferating cells with ITPA deficiency. Our results showed that cell growth arrest depends on p53 and MLH1 but not on ENDOV or MPG. However, this does not mean that ENDOV and MPG do not recognize dI in DNA. At first, we hypothesized that MLH1 contributes to the growth suppression of ITPA-deficient cells as a component of MMR. However, the growth arrest was independent of MSH2, an essential component of MMR, suggesting that MLH1 contributes to the growth suppression in an MMR-independent manner. *ITPA* KD induced SSBs in nuclear DNA and G1 phase cell cycle arrest in H414 cells, the mechanism of which may be different from the cell cycle arrest in G2 or S phase induced by MNNG or 5-FU. MLH1 is essential for both MMR and meiotic crossing-over in diploid germline cells. In the latter case, the MutS-γ complex, a heterodimer of MSH4 and MSH5, and the MutL-γ complex, a heterodimer of MLH1 and MLH3 play essential roles, however MSH2 complexes do not^60^. In addition, Siehler *et al*. reported MLH1-dependent and MSH2-independent suppression of homologous recombination by using MLH1- or MSH2-deficient human cell lines derived from colorectal adenocarcinoma^61^. Thus, our finding reveals another MMR-independent role of MLH1 in non-germline cells. The stabilization of p53 seems to play a central role in the ITPA deficiency-induced growth arrest. Previously, we observed S phase cell cycle arrest of HeLa MR cells after *NUDT16* KD^20^. Although degradation of p53 and Rb proteins are enhanced by HPV18 E6 and E7 proteins in HeLa MR cells, some chemical treatments stabilize p53 in HeLa cells^62^. Such reversible interference might cause the difference between HeLa MR and H414 cells after *NUDT16* or *ITPA* KD. Accumulated dITP may be recognized by DNA polymerases during DNA replication or DNA repair, and may stall the DNA polymerase complex. Repeated proofreading by the DNA polymerase is a candidate mechanism for polymerase stalling. Fujiwara H. *et al*. reported that Deep Vent polymerase (Exo^−^) but not Deep Vent (Exo^+^) could amplify DNA using dI-containing primers, suggesting that the proofreading function of some DNA polymerases may recognize a base pair of hypoxanthine and a normal base as a mismatch pair^63^. The stalled replication complexes or dI in DNA itself might induce SSBs in DNA dependent on MMR-independent function of the MLH1/PMS2 complex (Fig. 9) because our data indicate that MLH1 and PMS2 are required to induce SSBs in DNA (Figs 3A and 7B) and growth arrest (Figs 2B and 3A) under ITPA-deficient conditions. These findings indicate that the MLH1/PMS2 heterodimer functions as a DNA endonuclease in ITPA-deficient cells. Moreover, MLH1 is essential to keep higher basal p53 activity (Figs 8C and S4D) and induced levels of p53 protein and p21 mRNA/protein in H414 compared with HCT116 cells. Activation of p53 may depend on formation of SSBs in DNA, which may lead to undetectable levels of double-stand breaks, or depend on interaction between MLH1 and ATM as reported previously^64^,^65^. Induced p53 itself and/or its downstream effectors including p21 may cause G1 arrest. In our previous report using MLH1-proficient HCT116 + Chr3 and MLH1-deficient HCT116 cells, we observed higher *p21* mRNA levels in MLH1-proficient cells^66^. Cordycepin-treatment induced higher p21 protein levels in HCT116 + Chr3 cells compared with MLH1-deficient HCT116 + Chr2 cells^67^. In addition, several groups reported MLH1-dependent activation of p53^68^,^69^,^70^. It seems that an MLH1-dependent higher basal level of p53 activity and MLH1-dependent activation of p53 by ITPA-deficiency synergistically promote cell growth arrest in the ITPA-deficient cells. To elucidate these precise mechanisms, further study is necessary.

## Methods

### Animal Experiments

All animals were maintained in an air-conditioned, light/time-controlled, specific-pathogen-free room. All studies were approved by the Animal Care and Use Committee of Kyushu University. All protocols were performed in compliance with the “Fundamental Guidelines for Proper Conduct of Animal Experiment and Related Activities in Academic Research Institutions under the jurisdiction of the Ministry of Education, Culture, Sports, Science and Technology of Japan”.

### Oligonucleotides

Synthetic oligonucleotides are listed in Supplementary Table S3. All oligonucleotides were purchased from Applied Biosystems (Foster City, CA, USA), Integrated DNA technologies (Coralville, IA, USA) or Fasmac (Kanagawa, Japan).

### Cell Culture

Human fibroblast GM01617 (P32T) cells were obtained from the Coriell Institute (Camden, NJ, USA). WI38 and IMR90 cells were obtained from Japan Cancer Research Resources Bank (Tokyo, Japan). HCT116 cells were obtained from ATCC (Manassas, VA). A Mer^−^ variant of HeLaS3 strain, HeLa MR^71^, was kindly supplied by Dr. Rufus S. Day III, National Cancer Institute, NIH (Frederick, MD), and has been maintained in our laboratory. WI38, P32T, and IMR90 cells were routinely grown in minimum essential medium α (Life Technologies, Carlsbad, CA, USA). HeLa MR, HCT116 and H414 cells were grown in Dulbecco’s modified Eagle’s medium (DMEM; Life Technologies). Both media were supplemented with 10% heat-inactivated foetal bovine serum (FBS; PAA laboratories GmbH, Pasching, Austria), penicillin (100 U/ml), and streptomycin (100 μg/ml) (Life Technologies). These cell lines were cultured at 37 °C in a 5% CO_2_ atmosphere.

### Cell Proliferation Assay

Cell proliferation was analysed with a Cell Counting Kit-8, a colorimetric assay kit using WST-8 reagent (Dojindo, Kumamoto, Japan) according to the manufacturer’s instructions. This assay is similar to the 3-[4,5-dimethylthiazol-2-yl]-2,5-diphenyl-tetrazolium bromide (MTT) assay. Cells were seeded at a density of 500 cells per well in 96-well plates. After cultivation, WST-8 reagent was added to each well and cells were incubated for an additional 4 h. Absorbance of each sample was measured at 450 nm using an Infinite 200 Pro microplate reader (Tecan, Salzburg, Austria).

### Cell Cycle Analysis

To analyze the cell cycle, flow cytometric analysis of isolated nuclei samples was performed as previously described^20^ using a FACSCalibur, CellQuest (BD Biosciences, San Jose, CA, USA) and ModFit LT Version 3 software (Verity Software House, Topsham, ME, USA).

### Real-Time Quantitative RT-PCR

Total RNA from each cell culture was isolated with ISOGEN RNA extraction reagent (Nippon Gene, Tokyo, Japan). The relative levels of *ITPA, NUDT16, MPG, ENDOV, MLH1, CDKN1A (p21*), *TP53 (p53*) and *PMS2* mRNA were determined by real-time quantitative RT-PCR, according to previously described methods^20^,^72^. Briefly, 20 μg of each total RNA was treated with 10 units of RNase-free DNase I (Roche Applied Science, Penzberg, Germany) at 37 °C for 1 h, and then purified by phenol/chloroform treatment and ethanol precipitation. cDNA was synthesized from 2 μg of DNA-free total RNA using a high-capacity cDNA reverse transcription kit (Applied Biosystems) using random primers in a total volume of 20 μl. Real-time quantitative PCR was performed to measure the levels of each mRNA using a Thermal Cycler Dice^®^ Real-Time System Single (Takara, Kyoto, Japan) with 10 ng cDNA, 200 nM primers and Thunderbird^®^ SYBR^®^ qPCR Mix (Toyobo, Osaka, Japan) in a total volume of 25 μl. The specificity of the PCR products was established by dissociating curve analysis. No primer dimers were observed. The mRNA level of each gene was normalized to that of *18S rRNA*.

### Comet Assay

Nuclear DNA fragmentation was monitored using a Comet Assay Kit and Comet Assay Electrophoresis System II (Trevigen, Gaithersburg, MD, USA) under alkaline and neutral conditions, according to the manufacturer’s instructions. H414 and HCT116 cells were transfected with control or *ITPA* siRNA. After incubation for 4 days, the cells were separately embedded in soft agarose on glass slides and then subjected to the assay. Comet images from more than 30 cells for each siRNA were captured using an Axioskop 2 plus equipped with an AxioCam (Carl Zeiss MicroImaging Japan, Tokyo, Japan), and were analysed using the Comet Assay Software Project (CASP) program^73^ to quantify tail moment.

### Statistical Analysis

Statistical analyses were conducted using JMP 11.00 (SAS Institute, Cary, NC, USA). To assess statistical significance, we performed 1-way or 2-way analysis of variance (ANOVA); results obtained by standard least square fits are shown. For multiple comparison tests, ANOVA analyses were followed by a *post hoc* Tukey’s honestly significant difference (HSD) test. The Steel-Dwass test, Pearson’s chi-square test or Fisher’s exact test was applied for non-parametric multiple comparisons. The threshold *P* values for statistical significance were <0.05 (*), <0.01 (**), and <0.001 (***).

## Additional Information

**How to cite this article**: Yoneshima, Y. *et al*. Deoxyinosine triphosphate induces MLH1/PMS2- and p53-dependent cell growth arrest and DNA instability in mammalian cells. *Sci. Rep.* **6**, 32849; doi: 10.1038/srep32849 (2016).

## Supplementary Material

Supplementary Information
