# Supplementary material for: Deoxyinosine triphosphate induces MLH1/PMS2- and p53-dependent cell growth arrest and DNA instability in mammalian cells
Source: Sci Rep. 2016 Sep 13;6:32849. doi: 10.1038/srep32849 (PMC5020429; doi:10.1038/srep32849)
Supplement: Supplementary Information [file srep32849-s1.pdf]

Supplementary Information

Deoxyinosine triphosphate induces MLH1/PMS2- and p53-dependent cell growth arrest and DNA instability in mammalian cells

Yasuto Yoneshima[1,2], Nona Abolhassani[1], Teruaki Iyama[1], Kunihiko Sakumi[1,3], Naoko Shiomi[4], Masahiko Mori[4], Tadahiro Shiomi[4], Tetsuo Noda[5], Daisuke Tsuchimoto[1,3], Yusaku Nakabeppu[1,3]

[1]Division of Neurofunctional Genomics, Department of Immunobiology and Neuroscience, Medical Institute of Bioregulation, Kyushu University, Fukuoka 812-8581, Japan

[2]Research Institute for Diseases of the Chest, Graduate School of Medical Sciences, Kyushu University, Fukuoka 812-8581, Japan

[3]Research Center for Nucleotide Pool, Kyushu University, Fukuoka 812-8581, Japan

[4]National Institute of Radiological Sciences, Chiba 263-8555, Japan

[5]Cancer Institute, Japanese Foundation for Cancer Research, Tokyo 135-8550, Japan

SUPPLEMENTAL MATERIALS AND METHODS

Western Blot Analysis. Protein samples (10 μg protein/lane) were separated by sodium dodecyl sulphate polyacrylamide gel electrophoresis and transferred onto Immobilon-FL membranes (Millipore, Billerica, MA, USA). The membranes were incubated in Odyssey blocking buffer (LI-COR, Lincoln, NE, USA) containing anti-ITPA [1], anti-NUDT16 [2], anti-glyceraldehyde-3-phosphate dehydrogenase (GAPDH)(Millipore; MAB374) or anti-MLH1 (BD Biosciences; 551091) antibodies with appropriate fluorophore-conjugated secondary antibodies (LI-COR). Quantitative detection of fluorescent signals was performed using the Odyssey infrared imaging system (LI-COR). For the detection of check point related proteins, protein samples were separated, transferred onto 0.45 mm Immobilon-P membranes (Millipore), and sequentially treated with antibodies to phosphorylated CHK1(S345), CHK2(T68), p53(S15), p53(S33), or p38MAPK(T180/Y182) (Cell Signaling Technologies, Danvers, MA, USA) or with antibodies to p53 (Cell Signaling Technologies), p21 (BD biosciences,) or MDM2 (Santa Cruz Biotechnology, Dallas, TX, USA). Then, the membranes were treated with horseradish peroxidase-conjugated antibodies to mouse or rabbit IgG (Cell Signaling Technology). Chemiluminescent signals were detected with the ECL Prime Western Blotting Detection System (GE Healthcare Bio-Sciences, Piscataway, NJ, USA) and the EzCaptureMG image capture system (ATTO, Tokyo, Japan).

Nucleoside Treatment. HCT116 and H414 cells were treated with 2'-deoxyinosine or inosine (Sigma-Aldrich, St. Louis, MO, USA) as described below. The cells were transfected with siRNAs by electroporation and reseeded at a density of 500 cells per well in 96-well plates 24 h after the

transfection. After incubation for 1 hour, the medium was replaced with new medium containing nucleoside. The cultures were subjected to cell proliferation assays after incubation for a further 1, 2, 3, or 4 days.

siRNA and Transfection. All siRNA oligonucleotides used in this study, control siRNA#1 (Silencer Select Negative Control #1 siRNA, Cat#4390844) and control siRNA#2 (Silencer Negative Control #1 siRNA, Cat#AM4635), *NUDT16* siRNA (Silencer Select *NUDT16* siRNA; Cat#s43642), *ITPA* siRNA (Silencer Select *ITPA* siRNA; Cat#s7620), *MPG* siRNA (Silencer *MPG* siRNA; Cat#41190), *ENDOV* siRNA (Silencer Select *ENDOV* siRNA; Cat#s8930), *MLH1* siRNA (Silencer *MLH1* siRNA; Cat#119549), *TP53* (*p53*) siRNA (Silencer Select *TP53* siRNA; Cat#s605), *CDKN1A* (*p21*) siRNA (Silencer *CDKN1A* siRNA; Cat#s417), and *PMS2* siRNA (Silencer Select *PMS2* siRNA; Cat#s10740) were purchased from Applied Biosystems. HeLa MR, WI38, HCT116 and H414 cells were transfected with siRNAs by electroporation using a MicroPorator-Mini MP100 (Digital Bio Technology, Seoul, Korea), according to the manufacturer's instructions. In brief, 10^5 cells were suspended in 10 μl of R buffer (provided in the MicroPorator kit) and mixed with 1 μl of one of the siRNAs (50 μM) before electroporation. The transfected cells were suspended in fresh culture medium. For double knockdown of *ITPA* and *PMS2*, H414 cells were transfected with siRNAs using LipofectAmine RNAi Max reagent (Life Technologies, Carlsbad, CA, USA) to improve knockdown efficiency. After incubation for 24 h, the cells were reseeded for each assay. The estimated number of cell divisions between siRNA transfection and each assay was less than four. Knockdown efficiency was confirmed by real-time quantitative reverse-transcription polymerase chain reaction (RT-PCR) using total RNA prepared from cells 2 or 3 days after siRNA transfection (Supplementary Figure S2, S4).

Establishment of an MLH1-proficient H414 cell line. A human MLH1-proficient H414 cell line was established by targeted knock-in of wild-type exon 9 sequence into one allele of human *MLH1* in HCT116 cells. A 2.3 kbp DNA region including exon 7 and 8 of *MLH1* was amplified as a 5'-targeting element by PCR from genomic DNA of HeLa cells. *Sal*I and *Not*I recognition sites were added to its 5' and 3' ends, respectively, using a primer set for 5'-targeting element (Supplementary Table S3). Similarly, a 3.6 kbp DNA region including normal exon 9 of *MLH1* was amplified as a 3'-targeting element by PCR from HeLa cells, and *Not*I and *Asc*I sites were added to its 5' and 3' ends, respectively, using a primer set for 3'-targeting element. A promoter-less *Neo* expression cassette (IRES-Neo-pA) was amplified from pIRESneo2 (Clontech, Mountain View, CA, USA), and a *Not*I site and a loxP sequence, ATAACTTCGTATAGCATACATTATACGAAGTTAT, were added to both 5' and 3' ends of the *Neo* cassette. These DNA fragments were assembled in pBluescript II KS with a MC1DT-ApA cassette as described previously (Supplementary Figure S3A) [3]. The targeting vector was linearized by *Asc*I digestion and transfected into HCT116 cells by electroporation. For the selection of targeted cells, G418 was added to culture medium at 350 μg/ml. After 14 days, colonies were isolated and expanded. Genomic DNA from individual colonies was screened for gene targeting by PCR using the primer sets, P1-F/ P1-R and P2-F/ P2-R, and two targeted clones (clone 1 and 2) were obtained. We transfected a CRE recombinase expressing plasmid, pCXCre (a kind gift from Dr. Masaru Okabe, Osaka University), into these clones to delete the floxed Neo cassette. The transfected cells were cultured in medium with or without G418. A G418-sensitive stable clone derived from clone 1 was screened to be MLH1-proficient by sequence analysis of MLH1 exon 9, and by western blot analysis of MLH1 protein with a mouse anti-MLH1 antibody (Supplementary Figure S3B and C). We termed this

clone, H414.

Preparation of primary MEFs. *Itpa* and *Mlh1* KO mice were previously established as described [4,5]. The *Mlh1* KO mice were backcrossed to C57BL/6J mice for 10 generations by Professor Mutsuo Sekiguchi and Dr. Riyoko Ito from Fukuoka Dental College before this study. These KO mice were mated to generate double heterozygous mice ($Itpa^{+/-}/Mlh1^{+/-}$). Forty-two embryonic gestation day 14.5 (E14.5) embryos were obtained by intercross mating of the double heterozygous mice (11 pairs). Pregnant female mice were euthanized by cervical dislocation, and then the embryos were collected. Genotypes were analysed by PCR using tail DNA. PCR primers to detect *Itpa*-mutant, *Mlh1*-mutant, and wild-type alleles are described in Supplementary Table S3. Skin fibroblasts were aseptically isolated from at least five independent embryos of each genotype. Their genotypes are shown in Supplementary Table S4. These primary MEFs were cultured in DMEM supplemented with 10% heat-inactivated FBS, penicillin (100 units/ml), and streptomycin (100 μg/ml) at 37°C in a 5% CO_2 atmosphere. Primary MEFs were harvested by treatment with 0.15% trypsin–0.08% EDTA in phosphate-buffered saline and reseeded for further passage. Those from Passage 2 were stored in liquid nitrogen as primary MEFs.

Quantification of Deoxyinosine (dI) by Liquid Chromatography Coupled with Tandem Mass Spectrometry (LC-MS/MS). dI levels in DNA were determined as follows. The heads from 42 E14.5 embryos, obtained by intercrossing double heterozygous mice ($Itpa^{+/-}/Mlh1^{+/-}$) were stored at −80 °C. The preparation of nuclear DNA from these samples was performed using a DNA Extractor® TIS Kit (Wako Pure Chemical Industries, Osaka, Japan) according to the manufacturer's instructions, except

that 10 mM 2,2,6,6-tetramethylpiperidine-N-oxyl (TEMPO, Wako) and 20 μM 2'-deoxycoformycin, an adenosine deaminase inhibitor (Santa Cruz), were added to all reagents at all stages of manipulation, according to the method described by Taghizadeh *et al.*[6] The DNA samples were digested with Nuclease P1 (Wako) and acid phosphatase from potato (Sigma-Aldrich) in the presence of 20 mM TEMPO and 20 μM 2'-deoxycoformycin, and the digested samples were subjected to LC-MS/MS analysis using the Shimadzu VP-10 HPLC system (Shimadzu Corporation, Kyoto, Japan) connected to the API3000 MS/MS system (PE-SCIEX, Applied Biosystems), as described previously [7]. To analyze dI in DNA from H414 cells, we exactly followed Taghizadeh's protocol [6] and used Shimazu Nexera UHPLC/HPLC system (Shimazu Croporation, Kyoto, Japan) connected to the API3200 MS/MS system (AB-SCIEX, Applied Biosystems).

Establishment of MSH2-deficient cell lines. MSH2-deficient H414 cell lines were established using the CRISPR/Cas9 system with pScCas9(BB)-2A-Puro (pX459) (Addgene ID: 48139) [8]. A target sequence, ACATTTATCAGGACCTCAAC, was designed in exon 4 of human MSH2 transcript 1 using the CRISPR Design Tool (http://crispr.mit.edu/). A targeting plasmid expressing Cas9 and single-guide RNA for the target sequence was prepared by inserting annealed sense and antisense oligo-DNAs for gRNA (Table S3) into the *Bbs*I site as described by Ran *et al.* [8]. H414 cells were transfected with the targeting plasmid, and cultured for 24 h. Then, the cells were reseeded and cultured in the presence of 0.5 μg/ml puromycin (Sigma-Aldrich, St. Louis, MO, USA) for 24 h to remove untransfected cells. The resulting colonies formed were isolated 2 weeks later. The DNA fragment containing the target sequence was amplified from genomic DNA by PCR with primers MSH2Ex5Fw/MSH2Ex5Rv (Table S3). The PCR products were analyzed for their sensitivity to

*Eco*O109I (Life Technologies, Carlsbad, CA, USA), a recognition site for which exists in the target sequence, and then subjected to sequencing analysis. The cell lines with two mutant MSH2 alleles were analyzed for MSH2 protein by western blot analysis with rabbit antibodies against theN-terminal 20 amino acids of human MSH2 (Santa Cruz Biotechnology, Dallas, TX, USA) and the C-terminal 271 amino acids of mouse MSH2 (mMSH2-CT). The antibody against mMSH2-CT was prepared as shown below. First, we prepared rabbit antiserum against a fusion protein of *Escherichia coli* TrpE and mMSH2-CT (TrpE-mMSH2-CT), as described previously [9]. Specific antibodies were purified with the aid of TrpE-mMSH2-CT–Sepharose and TrpE–Sepharose columns [9,10].

***In Situ* Nick Translation.** *In situ* nick translation was performed according to the protocol by Trimarchi *et al.* [11]. Three days after siRNA transfection, H414 cells were harvested, and fixed in 3:1 (v/v) methanol:acetic acid. The fixed cells were spread and dried on glass slides. The slides were immersed for an hour in 1x PBS and later rinsed in distilled water. Each slide was incubated with reaction mix [0.1 U/µl DNA polymerase I (Takara), 2% (v/v) DIG-DNA labeling mix (Roche Applied Science), 50 mM Tris-HCl pH 7.8, 10 mM $MgCl_2$, 0.1 mM DTT, and 0.0025% BSA] at 37°C for an hour in a moist chamber. Slides were immersed for 5 min in 1% trichloroacetic acid solution at 4°C to remove unincorporated dNTPs and later rinsed in buffer I [1M Tris-HCl, 1M NaCl, 0.04M $MgCl_2$, 0.5% (v/v) Triton X-100, final pH 7.5]. Slides were then incubated for 20 min in buffer II [2% (w/v) bovine serum albumin in buffer I] at 42°C to stop the reaction. After washing slides in buffer I, they were incubated for an hour in detection solution [0.4 µg/ml anti-DIG-Fluorescein Fab fragments (Roche Applied Science), 10% (v/v) buffer II in deionized H_2O] in a moist chamber at 37°C in the dark. After washing in buffer I and 1x PBS, slides were incubated for 4 min in 1µg/ml propidium iodide solution at room

temperature in the dark. Slides were rinsed in 1x PBS and dehydrated through an alcohol series. After mounting, the slides were observed using an Axioskop 2 plus microscope equipped with AxioCam and AxioVision software (Carl Zeiss MicroImaging Japan, Tokyo, Japan).

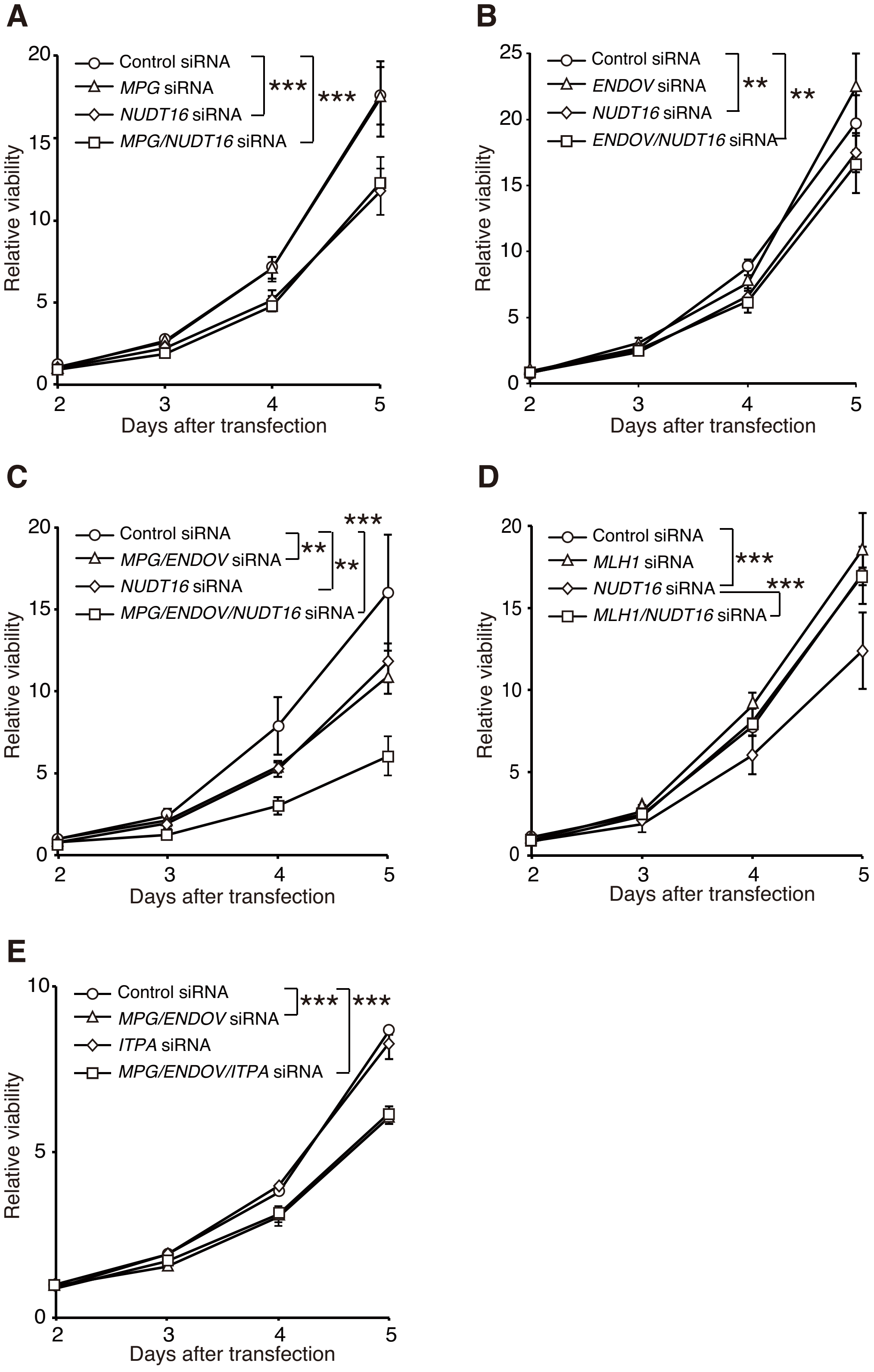
A
Control siRNA
MPG siRNA
NUDT16 siRNA
MPG/NUDT16 siRNA
***
***
Relative viability
Days after transfection
B
Control siRNA
ENDOV siRNA
NUDT16 siRNA
ENDOV/NUDT16 siRNA
**
**
Relative viability
Days after transfection
C
Control siRNA
MPG/ENDOV siRNA
NUDT16 siRNA
MPG/ENDOV/NUDT16 siRNA
**
**
***
Relative viability
Days after transfection
D
Control siRNA
MLH1 siRNA
NUDT16 siRNA
MLH1/NUDT16 siRNA
***
***
Relative viability
Days after transfection
E
Control siRNA
MPG/ENDOV siRNA
ITPA siRNA
MPG/ENDOV/ITPA siRNA
***
***
Relative viability
Days after transfection

Supplementary Figure S1. Knockdown of *MLH1* rescues cell growth delay caused by *NUDT16* knockdown in HeLa MR cells.

(A) HeLa MR cells transfected with control, *MPG* and/or *NUDT16* siRNA. Results tested with two-way ANOVA, P<0.001; Tukey's HSD *post hoc* test, P<0.001 (control versus *NUDT16* or control versus *MPG/NUDT16* siRNA). (B) HeLa MR cells transfected with control, *ENDOV* and/or *NUDT16* siRNA. Results tested with two-way ANOVA, p<0.001; Tukey's HSD *post hoc* test, P=0.0031 (control versus *NUDT16* siRNA), P=0.0018 (control versus *ENDOV*/*NUDT16* siRNA). (C) HeLa MR cells transfected with control, *NUDT16 MPG* and/or *ENDOV* siRNA. Results tested with two-way ANOVA, P<0.001; Tukey's HSD *post hoc* test, P=0.0014 (control versus *MPG*/*ENDOV* siRNA), P=0.001 (control versus *NUDT16* siRNA), P<0.001 (control versus *MPG/ENDOV*/*NUDT16* siRNA). (D) HeLa MR cells transfected with control, *MLH1* and/or *NUDT16* siRNA. Results tested with two-way ANOVA, P<0.001; Tukey's HSD *post hoc* test, P<0.001 (control versus *NUDT16*), P<0.001 (*NUDT16* versus *MLH1/NUDT16*). (E) HeLa MR cells transfected with control, *MPG, ENDOV* and/or *ITPA* siRNA. Results tested with two-way ANOVA, P<0.001; Tukey's HSD *post hoc* test, P<0.001 (control versus *MPG/ENDOV* or *MPG/ENDOV/ITPA*). Data are presented as the mean±SD (n=3).

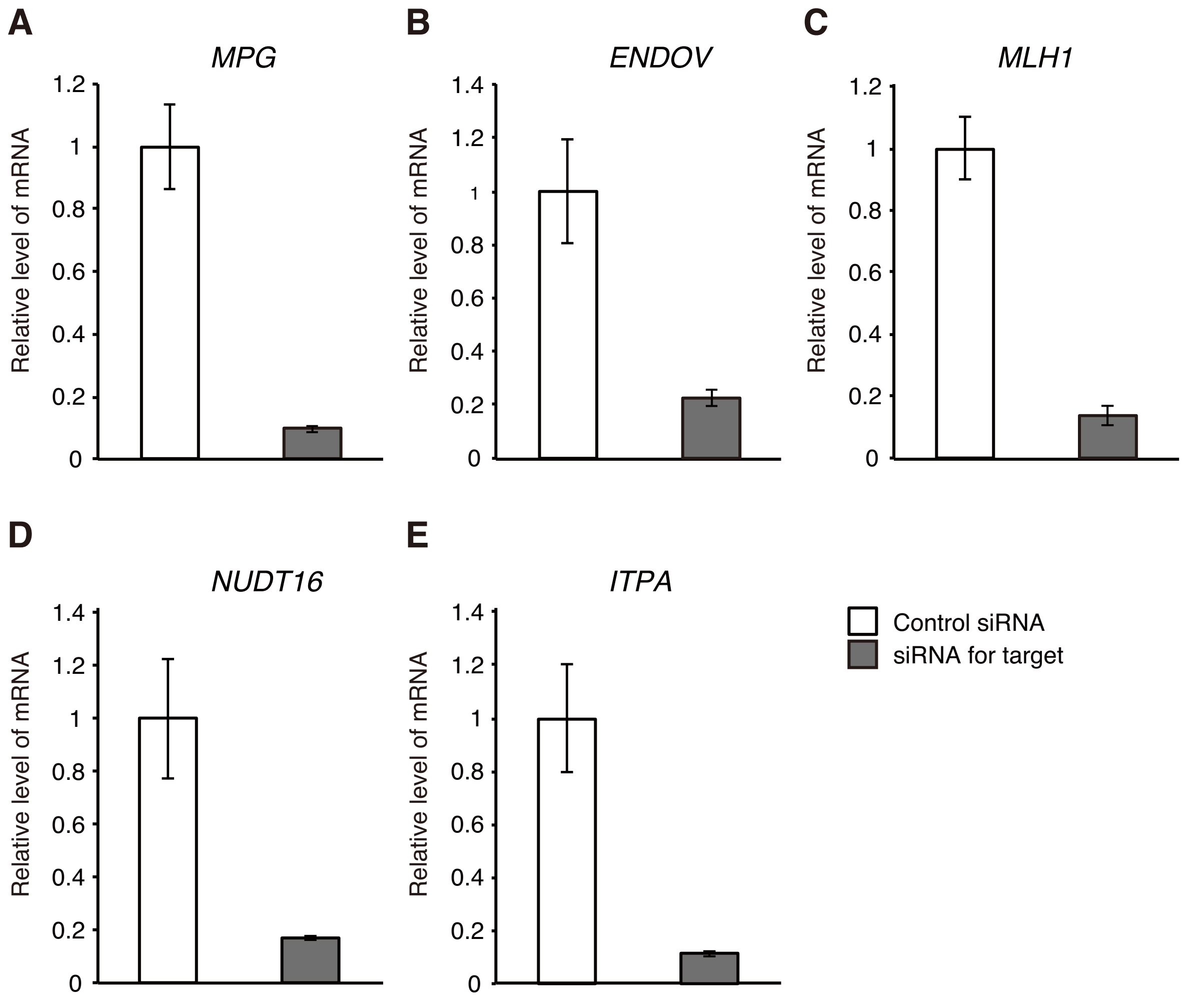

Supplementary Figure S2. Knockdown efficiency of siRNAs in HeLa MR cells.

(A-E) *MPG*, *ENDOV*, *MLH1*, *NUDT16* and *ITPA* mRNA levels in HeLa MR cells transfected with siRNA for each gene and/or control siRNA were analysed. Two days after siRNA transfection, RNA levels were analysed by real time quantitative RT-PCR. Data for each mRNA was normalized to that of 18S rRNA. The levels relative to the control cells transfected with siRNA are shown as the mean±SD (n=3).

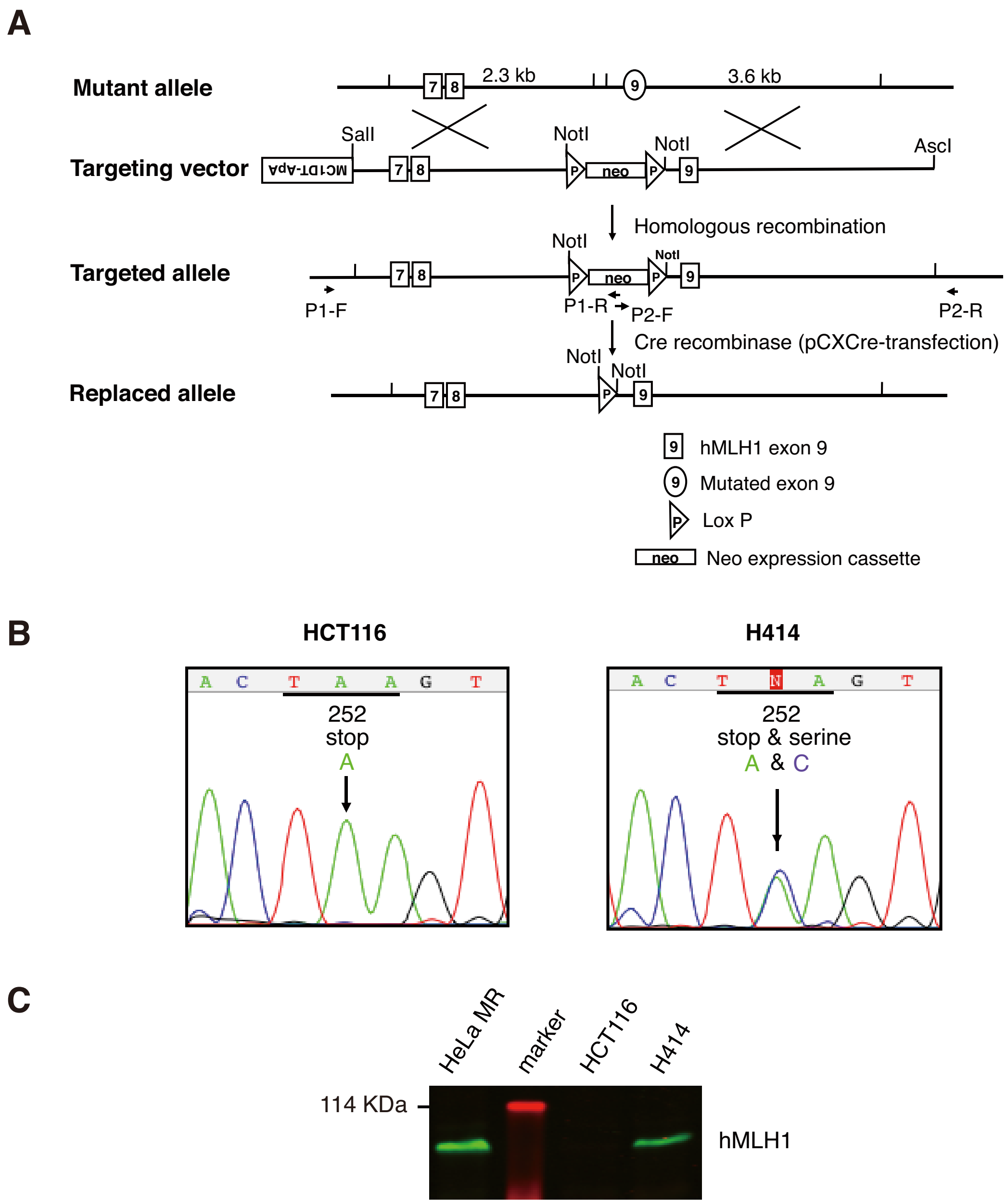

Supplementary Figure S3. Establishment of H414 cell line.

(A) Schematic representation of knock-in targeting the *MLH1* locus. Configurations of the original mutant allele, targeting construct, targeted allele and replaced allele after Cre-treatment are shown. Relevant restriction sites and the positions of the primers used for PCR analysis are shown. MC1DT-ApA, diphtheria toxin gene; neo, neomycin resistance gene; p, Lox P site. Wild-type exons 7, 8 and 9 are indicated by numbered boxes. Mutant exon 9 is indicated by the numbered circle. (B)

Sequence analysis of *MLH1* exon 9 in genomic DNA from HCT116 and H414 cells. (C) Western blot analysis of MLH1 in whole-cell extracts from H414 cells. HeLa MR and HCT116 cells were used as positive and negative controls, respectively.

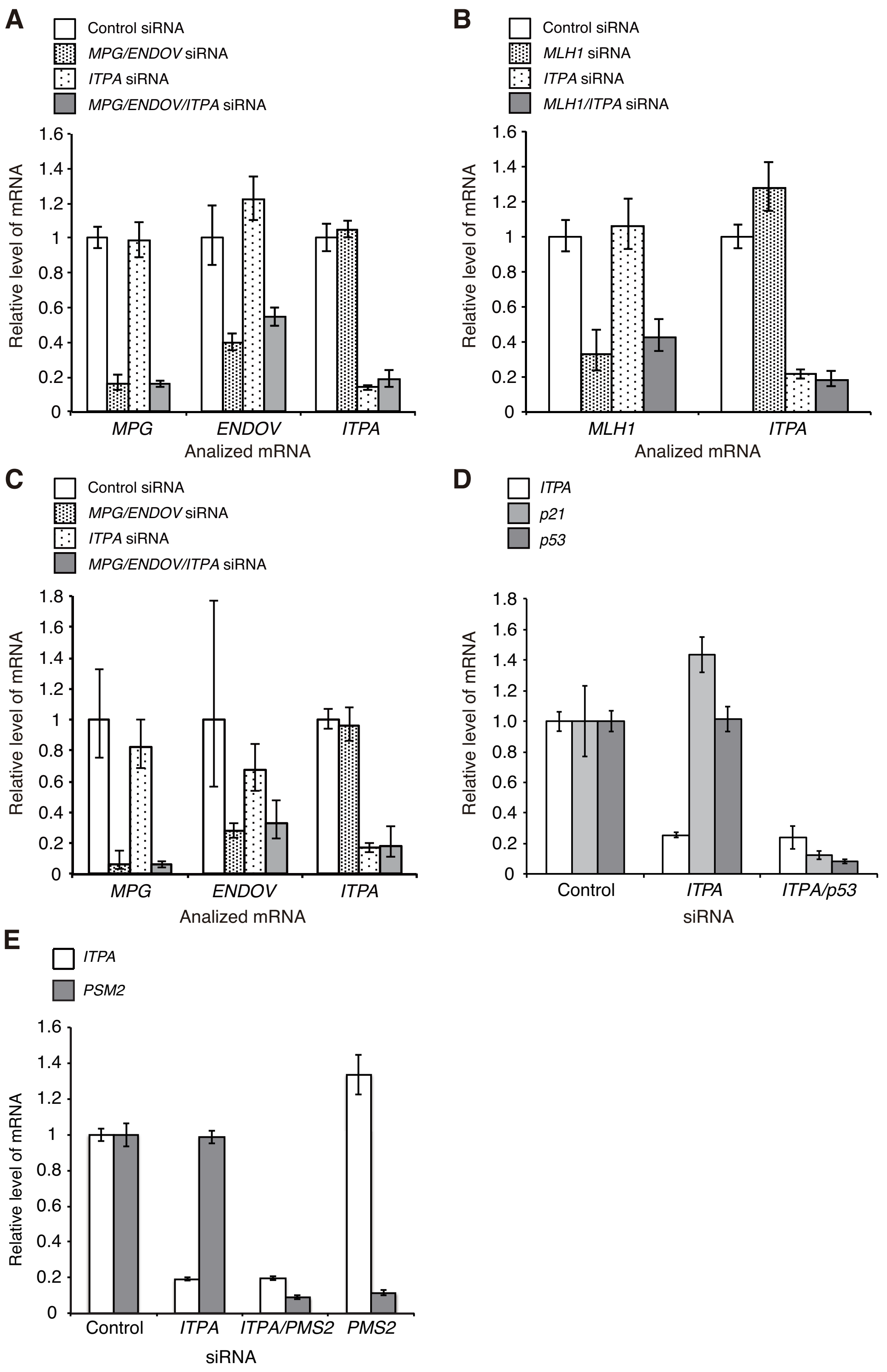

Supplementary Figure S4. Knockdown efficiency of siRNAs in H414 and HCT116 cells.

(A) *MPG*, *ENDOV* and *ITPA* mRNA levels in H414 cells transfected with each siRNA. (B) *MLH1* and

ITPA mRNA levels in H414 cells transfected with each siRNA. (C) *MPG*, *ENDOV* and *ITPA* mRNA levels in HCT116 cells transfected with each siRNA. (D) *ITPA*, *p53* and *p21* mRNA levels in H414 cells transfected with negative control, *ITPA* or *ITPA* plus *p53* siRNA. (E) *ITPA* and *PMS2* mRNA levels in H414 cells transfected with negative control, *ITPA*, *PMS2* and *ITPA* plus *PMS2* siRNA. Two days after siRNA transfection, RNA levels were analysed by real time quantitative RT-PCR. Data for each mRNA was normalized to that of 18S rRNA. The levels relative to those in control cells transfected with siRNA are shown as the mean±SD (n=3). Induction of *p21* mRNA 2 days after *ITPA* siRNA treatment was less than the induction 3 days after the treatment shown in Figure 8B.

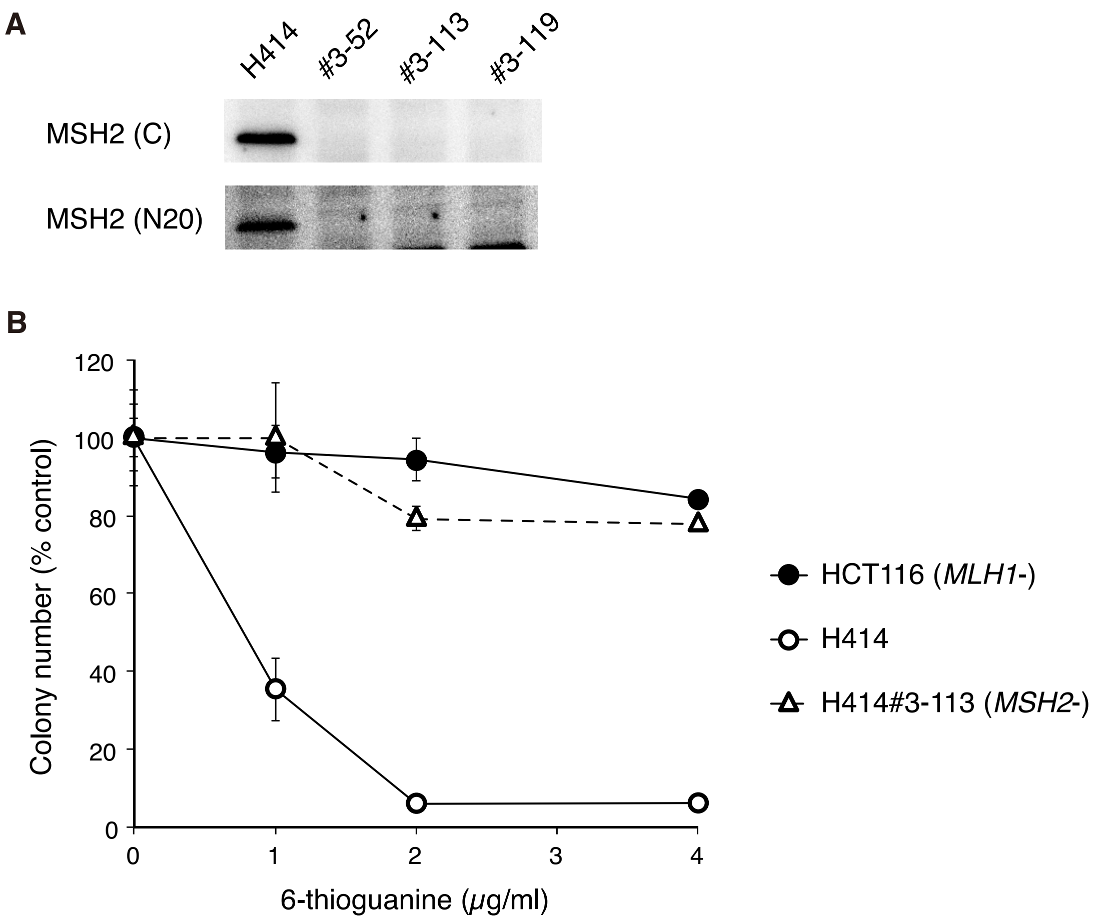

Supplementary Figure S5. Establishment of MSH2 knockout clones from H414 cells.

(A) Western blot analysis of MSH2 in whole cell extracts. Ten micrograms of protein from each of three KO clones and H414 cells were separated by 10% SDS-PAGE, and subjected to western blot with

antibodies against the C- or N-terminal region of human MSH2. (B) MSH2 KO clone #3-113, MMR-proficient H414, and MLH1-deficient HCT116 cells were analysed for their sensitivities to 6-thioguanine (6-TG). After incubation with 6TG for 24h, the cells were cultured for 10 days to allow colony formation. Results were tested with two-way ANOVA, $P<0.001$; Tukey's honestly significant difference (HSD) *post hoc* test, $P<0.0001$ (H414 versus HCT116), $P<0.0001$ (H414 versus H414#3-113).

Supplementary Table S1. Genotype of offspring mice obtained from cross-mating of double hetero mice (*Itpa*$^{+/-}$/*Mlh1*$^{+/-}$)

Genotype		*Mlh1*			Ratio		
		+/+	+/-	-/-	+/+	+/-	-/-
Itpa	+/+	9	11	2	0.12	0.15	0.03
	+/-	12	30	10	0.16	0.41	0.14
	-/-	0	0	0	0	0	0

Supplementary Table S2. Mutant *MSH2* alleles of the MSH2-deficient clones

Allele and clone nos	Target and flanking sequences in exon 4 of *MSH2* isoform 1
Wild-type allele	GACATTTATCAGGACCTCAACCGGTTG
Allele 1 of #3-52	GACATTT----------CAACCGGTTG
Allele 2 of #3-52	GACATTTATCAGGACCTCtaatcAACCGGTTG
Allele 1 of #3-113	G-----------------------TTG
Allele 2 of #3-113	GACATTTgtctatttt--------TTG
Allele 1 of #3-119	GACATTTATCAGGACCTCaAACCGGTTG
Allele 2 of #3-119	GACATTTATCAGGACCTC---- (from 4147 to 4715 of PX459) ----G

Capital letters: wild-type sequence

Lower case letters or parentheses: insertion

Hyphen: deletion

Supplementary Table S3. Synthetic oligonucleotides

Oligomer:	5'→3' sequence:	Description:
1	CCTGTCGACAAACCAGACCTTTGGGACACTCTG	Primer for 5'-targeting element of *hMLH1*
2	AGTAATGCGGCCGCTGAGTGTTCAACGGAGGGTACGGCTTG	Primer for 5'-targeting element of *hMLH1*
3	TAAACAGCGGCCGCAACAACACACATTGTAGAACCACGTTG	Primer for 3'-targeting element of *hMLH1*
4	TTCTATGGCGCGCCTCAAGGAAGTTGATTCTACCAGACGA	Primer for 3'-targeting element of *hMLH1*
5	AATGTCGACTGCTAGACACAATCCTTCGGTTC	P1-F
6	ATTTTCCACCATGATATTCGGCAAGCAGGC	P1-R
7	GCCTGCTTGCCGAATATCATGGTGGAAAAT	P2-F
8	GGGACAGGCGCGCCTTTTGCCAGTGGTGGTGTATGGGATTCACTC	P2-R
9	GCTTTCAGCCTGATGGATATGAG	Primer of real-time RT-PCR for *ITPA*
10	CGATGGGAGACAGCGTTCTT	Primer of real-time RT-PCR for *ITPA*
11	GACTGCTGGAGTCTGGCTCTA	Primer of real-time RT-PCR for *NUDT16*
12	GGCTGCCTCTAGTGATGAG	Primer of real-time RT-PCR for *NUDT16*
13	ACCACTCCGGGCCCATA	Primer of real-time RT-PCR for *MPG*
14	CAACCCCAGTCGGGTAAGGT	Primer of real-time RT-PCR for *MPG*
15	TTACAGACCTGGGGTGTGTGTTG	Primer of real-time RT-PCR for *ENDOV*
16	GGAGTCGGATCTTCTCCTTGTG	Primer of real-time RT-PCR for *ENDOV*
17	TGGGACGAAGAAAAGGAATG	Primer of real-time RT-PCR for *MLH1*
18	TCCAGGAGTTTGGAATGGAG	Primer of real-time RT-PCR for *MLH1*
19	GAACAGCTTTGAGGTGCGTG	Primer of real-time RT-PCR for *TP53*
20	TTGGGCAGTGCTCGCTTAG	Primer of real-time RT-PCR for *TP53*
21	CCCGTGAGCGATGGAACT	Primer of real-time RT-PCR for CDKN1A
22	CGCTCCCAGGCGAAGTC	Primer of real-time RT-PCR for CDKN1A
23	AGGTGTGGCTTATGGGCCCAGT	Genotyping for *Itpa* wild type
24	AGGCAGTCCCCCAAGTGCGT	Genotyping for *Itpa* wild type
25	GCGGTGAAGGTCAACGGGGGTT	Genotyping for *Itpa* knockout
26	TCGGGTCCTCCGGAGCGGGTT	Genotyping for *Itpa* knockout
27	AAGAAGAAAGCGGAGATGCTTGCAGAC	Genotyping for *Mlh1* wild type
28	GATAGATACATGCTGCTTCTGAGGGGA	Genotyping for *Mlh1* wild type
29	GAACAGTCTGAGCGTGAAGGTTTCATG	Genotyping for *Mlh1* knockout
30	CCTGAAGAACGAGATCAGCAGCCTC	Genotyping for *Mlh1* knockout
31	CACCGACATTTATCAGGACCTCAAC	Sense for gRNA
32	AAACGTTGAGGTCCTGATAAATGTC	Antisense for gRNA
33	TCTTATTCCTTTTCTCATAGTAGT	MSH2Ex5Fw
34	TCCATGTACCTGATTCTCCA	MSH2Ex5Rv
35	AGTCAGCGTGCAGCAGTTAT	PMS2 Fw
36	TGCAACTTACACGGATGCCT	PMS2 Rv

Supplementary Table S4. Genotypes of embryos obtained by intercross mating of *Itpa*$^{+/-}$/*Mlh1*$^{+/-}$ mice

Genotype		*Mlh1*		
		+/+	+/-	-/-
Itpa	+/+	5	11	5
		4 : 1	7 : 4	2 : 3
	+/-	9	20	13
		4 : 5	8 : 12	3 : 10
	-/-	5	10	5
		2 : 3	5 : 5	2 : 4

Upper: total number

Lower: sex (male : female)

(grey shading) Used for cell counting

REFFERENCES

1 Behmanesh, M. *et al.* Characterization of the structure and expression of mouse Itpa gene and its related sequences in the mouse genome. *DNA Res* **12**, 39-51 (2005).

2 Iyama, T., Abolhassani, N., Tsuchimoto, D., Nonaka, M. & Nakabeppu, Y. NUDT16 is a (deoxy)inosine diphosphatase, and its deficiency induces accumulation of single-strand breaks in nuclear DNA and growth arrest. *Nucleic Acids Res* **38**, 4834-4843, doi:10.1093/nar/gkq249 (2010).

3 Katsube, T. *et al.* Differences in sensitivity to DNA-damaging Agents between XRCC4- and Artemis-deficient human cells. *J Radiat Res* **52**, 415-424, doi:10.1269/jrr.10168 (2011).

4 Kawate, H. *et al.* Separation of killing and tumorigenic effects of an alkylating agent in mice defective in two of the DNA repair genes. *Proc Natl Acad Sci USA* **95**, 5116-5120 (1998).

5 Behmanesh, M. *et al.* ITPase-deficient mice show growth retardation and die before weaning. *Cell Death Differ* **16**, 1315-1322, doi:10.1038/cdd.2009.53 (2009).

6 Taghizadeh, K. *et al.* Quantification of DNA damage products resulting from deamination, oxidation and reaction with products of lipid peroxidation by liquid chromatography isotope dilution tandem mass spectrometry. *Nat Protoc* **3**, 1287-1298, doi:10.1038/nprot.2008.119 (2008).

7 Abolhassani, N. *et al.* NUDT16 and ITPA play a dual protective role in maintaining chromosome stability and cell growth by eliminating dIDP/IDP and dITP/ITP from nucleotide pools in mammals. *Nucleic Acids Res* **38**, 2891-2903, doi:10.1093/nar/gkp1250 (2010).

8 Ran, F. A. *et al.* Genome engineering using the CRISPR-Cas9 system. *Nat Protoc* **8**, 2281-2308, doi:10.1038/nprot.2013.143 (2013).

9 Nakabeppu, Y. & Nathans, D. A naturally occurring truncated form of FosB that inhibits Fos/Jun transcriptional activity. *Cell* **64**, 751-759 (1991).

10 Nakabeppu, Y., Oda, S. & Sekiguchi, M. Proliferative activation of quiescent Rat-1A cells by delta FosB. *Mol Cell Biol* **13**, 4157-4166 (1993).

11 Trimarchi, C., La Sala, D., Zamparelli, A. & Cinti, C. Detection of apoptotic deoxyribonucleic acid break by in situ nick translation. *Methods Mol Biol* **285**, 113-118, doi:10.1385/1-59259-822-6:113 (2004).
